# Information and communication technology-based interventions for suicide prevention implemented in clinical settings: a scoping review

**DOI:** 10.1186/s12913-023-09254-5

**Published:** 2023-03-23

**Authors:** Hwayeon Danielle Shin, Keri Durocher, Lydia Sequeira, Juveria Zaheer, John Torous, Gillian Strudwick

**Affiliations:** 1 Institute of Health Policy, Management and Evaluation, University of Toronto, Toronto, Ontario Canada; 2 Campbell Family Mental Health Research Institute, Centre for Addiction and Mental Health, Toronto, Ontario Canada; 3 Arthur Labatt Family School of Nursing, Western University, London, Ontario Canada; 4 School of Health, Community Service & Creative Design, Lambton College, Sarnia, Ontario Canada; 5 Health Outcomes and Performance Evaluation (HOPE) Research Unit, Institute for Mental Health Policy Research, Centre for Addiction and Mental Health, Toronto, Ontario Canada; 6 Gerald Sheff and Shanitha Kachan Emergency Department, Centre for Addiction and Mental Health, Toronto, Ontario Canada; 7 Department of Psychiatry, University of Toronto, Toronto, Ontario Canada; 8 Department of Psychiatry, Beth Israel Deaconess Medical Center, Harvard Medical School, Boston, Massachusetts USA

**Keywords:** Information communication technology, eHealth, Suicide prevention, Implementation, Digital health, Health Informatics, Psychiatry, Mental health

## Abstract

**Background:**

A large number of information and communication technology (ICT) based interventions exist for suicide prevention. However, not much is known about which of these ICTs are implemented in clinical settings and their implementation characteristics. In response, this scoping review aimed to systematically explore the breadth of evidence on ICT-based interventions for suicide prevention implemented in clinical settings and then to identify and characterize implementation barriers and facilitators, as well as evaluation outcomes, and measures.

**Methods:**

We conducted this review following the Joanna Briggs Institute methodology for scoping reviews. A search strategy was applied to the following six databases between August 17–20, 2021: MEDLINE, Embase, CINAHL, PsycINFO, Web of Science, and Library, Information Science and Technology Abstracts. We also supplemented our search with Google searches and hand-searching reference lists of relevant reviews. To be included in this review, studies must include ICT-based interventions for any spectrum of suicide-related thoughts and behaviours including non-suicidal self-injury. Additionally, these ICTs must be implemented in clinical settings, such as emergency department and in-patient units. We used the Preferred Reporting Items for Systematic Reviews and Meta-Analyses Extension for Scoping Reviews (PRISMA-ScR) checklist to prepare this full report.

**Results:**

This review included a total of 75 citations, describing 70 studies and 66 ICT-based interventions for suicide prevention implemented in clinical settings. The majority of ICTs were computerized interventions and/or applications (*n* = 55). These ICTs were commonly used as indicated strategies (*n* = 49) targeting patients who were actively presenting with suicide risk. The three most common suicide prevention intervention categories identified were post-discharge follow-up (*n* = 27), screening and/or assessment (*n* = 22), and safety planning (*n* = 20). A paucity of reported information was identified related to implementation strategies, barriers and facilitators. The most reported implementation strategies included training, education, and collaborative initiatives. Barriers and facilitators of implementation included the need for resource supports, knowledge, skills, motivation as well as engagement with clinicians with research teams. Studies included outcomes at patient, clinician, and health system levels, and implementation outcomes included acceptability, feasibility, fidelity, and penetration.

**Conclusion:**

This review presents several trends of the ICT-based interventions for suicide prevention implemented in clinical settings and identifies a need for future research to strengthen the evidence base for improving implementation. More effort is required to better understand and support the implementation and sustainability of ICTs in clinical settings. The findings can also serve as a future resource for researchers seeking to evaluate the impact and implementation of ICTs.

**Supplementary Information:**

The online version contains supplementary material available at 10.1186/s12913-023-09254-5.

## Introduction

The World Health Organization (WHO) reports that there are over 700,000 annual deaths by suicide worldwide [1, 2]. Globally, suicide is the fourth leading cause of deaths for youth and young adults [1], and specifically it is the second in Canada and USA [3, 4], and the first in Australia [5]. As such, suicide prevention is a top global health priority [6]. Suicide is preventable with timely, evidence-based interventions [2]. There are evidence-based interventions for suicide prevention, such as risk assessment, safety planning interventions, and follow-up care [7, 8], all of which are delivered in clinical settings, such as emergency departments. We also recognize the importance of population-level approaches to suicide prevention, such as gatekeeper training programs in schools [8]. However, clinical attention for suicide prevention cannot be overlooked, and individuals who suffer from suicide ideation must receive clinical attention [9, 10].

A review published in 2002 investigated 40 studies from the United States (US), United Kingdom (UK), Canada, Finland, and Sweden and found that 33% of individuals who died of suicide had contact with mental health services in the year before death and 20% in the month before death [11]. Not much has changed since then, and we continue to observe missed opportunities. In Canada, a study in 2014 examined 8,851 suicide deaths and found 50% of these individuals had visited an emergency department in the year before death, and one third had died within the month of discharge [12]. This speaks to a critical opportunity for suicide prevention in clinical settings, which will be the focus of this review.

Information and communication technology (ICT) [13] or eHealth [14] includes a wide range of digital tools such as internet, telemedicine, and mobile technologies. In this review, we refer to ICTs collectively as technologies, advanced multimedia, software programmes and/or telecommunications that allows efficient communication, management, storage, dissemination and exchange of information [13], and eHealth refers to use of ICTs for health [14]. There is a large number of ICT-based interventions for mental health, including suicide prevention strategies [15, 16]. For example, Rassy et al. identified 115 ICT-based interventions for suicide prevention, and they include web-based tools, online programs, and mobile applications [16]. Given the widespread use of technologies in this modern world, including mobile phones, ICTs have the potential to improve suicide prevention by removing geographical barriers and increasing access and availability of evidence-based interventions [16]. Additionally, ICTs may not replace clinical encounters, but it can be augmented to expand existing suicide prevention care.

There is a growing body of evidence for the effectiveness of ICT-based interventions for suicide prevention [15, 17–21]. For example, Witt et al. identified 14 online programs and mobile apps for self-management of suicide ideation and concluded with some evidence of reductions in suicidal ideation associated with using these digital interventions [20]. Arshad et al. also identified 22 clinical trials of ICT-based interventions for suicide prevention, which included online support tools for coping skills often derived from a well-established cognitive behavioural therapy and concluded with a favourable effect on reducing suicide thoughts [19]. Despite the clinical potential and a large number of available ICTs for mental health, clinical integration remains limited, and clinicians, service users, and hospitals continue to face challenges to achieve sustainable adoption [22–24]. It has been repeatedly reported that implementation of ICTs rarely moves beyond feasibility trials or initial adoption, and sometimes ICTs are abandoned [25].

Healthcare is a complex adaptive system, which is shaped by multiple, constant interdependent interactions and relationships [26, 27]. When complexities exist related to care settings or implementation challenges, the less likely ICTs are to be adopted and sustained [25, 28]. As such, research teams are required to move beyond traditional cause-and-effect thinking, embrace complexity, and examine dynamic processes inherent within. Specifically for mental health apps, there was a recent call for attention to complex contexts in which apps are being implemented [22]. It is critical to prospectively assess determinants of implementation and then strategically develop implementation strategies to match the contextual needs.

Efforts are needed to support clinical integration of ICT-based interventions for suicide prevention as well as their spread and maintenance to ensure that useful ICTs are reaching people who are in need. Currently, the literature on ICT-based interventions for suicide prevention describes their characteristics and/or evaluates their effectiveness in reducing suicide behaviours and risks, but not much is known about clinical integration of ICTs [15–20]. For example, it remains unknown how many of 115 ICT-based interventions for suicide prevention identified by Rassy et al. have been implemented in clinical settings [16]. Research has not yet comprehensively explored evidence on ICTs implemented in clinical settings and their implementation characteristics, including barriers and facilitators. Given the lack of successful clinical integration of ICTs [22–24], this review was needed as a first step to inform implementation efforts for useful ICTs for suicide prevention in clinical settings. Scoping reviews are suggested when researchers need to identify the types of available evidence and key characteristics related to a concept rather than to perform a meta-analysis to make practice recommendations [29, 30]. Furthermore, this review was needed to determine the range of studies before carrying out our future multi-phase project to develop and evaluate implementation strategies for a mobile app-based intervention for suicide prevention in clinical settings. As such, the current scoping review aimed to systematically explore the breadth of evidence on ICT-based interventions for suicide prevention implemented in clinical settings and then to characterize barriers and facilitators to implementation, as well as measures and outcomes reported in this body literature.

### Research questions

To achieve the research aim stated above, this scoping review addressed the following questions.

- 1. What ICT-based interventions for suicide prevention have been implemented in clinical settings?
- 1.1. What are the reported barriers and facilitators to implementing these ICT-based interventions?
- 1.2. What are the reported measures and outcomes of these ICT-based interventions?

## Methods

This review followed the Joanna Briggs Institute (JBI) methodology [31, 32] and this report was prepared following the Preferred Reporting Items for Systematic reviews and Meta-Analyses extension for Scoping Reviews (PRISMA-ScR) checklist [33]. Our a priori protocol has been previously published [34]. We also searched PROSPERO, the Cochrane Database of Systematic Reviews and JBI Evidence Synthesis and Open Science Framework in June-July 2021 and identified no ongoing systematic or scoping reviews on the same topic.

### Inclusion/exclusion criteria

#### Population

All clinicians both licenced and regulated practitioners were considered for inclusion in this review. Various healthcare professionals, such as physicians, nurses and social workers, provide direct care, and they are often collectively referred to as ‘clinicians’ [35, 36]. Additionally, unregulated clinical support team members and peer support workers were considered for inclusion because these roles are increasingly integrated into mental health care settings [37, 38]. This population criterion was relatively less significant than the context criterion because who implemented ICT-based interventions was often part of the context.

#### Concept

This review considered all types of ICT-based interventions for suicide prevention. Routine care (i.e., treatment as usual) provided via virtual platforms or telephones were excluded unless an ICT-based intervention was delivered to patients. Therefore, following the WHO’s definition for intervention, ICT-based interventions needed to assess, improve or promote service users’ health outcomes [39]. Suicide-related thoughts and behaviours is an umbrella term that refers to a spectrum of suicide ideation, communication, behaviours, and attempts with having any frequencies of suicidal thoughts with actual, undetermined, or no suicidal intent [40]. To be included, ICT-based interventions must be related to any sub-category of suicide-related thoughts and behaviours including non-suicidal self-injury (NSSI). Although NSSI is a unique phenomenon from actual suicide attempt, we decided to include it because NSSI is one of the risk factors for future attempt and suicide [41, 42]. According to WHO, there are three levels of suicide prevention: 1) Universal, 2) Selective, 3) Indicated. Universal strategies for suicide prevention work at a population level [1]. Selective prevention strategies target individuals who may not be currently expressing suicidal behaviours but are at a greater risk of suicide based on their characteristics such as age, sex and/or medical history [1]. Indicated prevention strategies target individuals who are presenting active risk or early signs of suicide potential, such as suicide attempt [1]. ICT-based interventions for any levels of suicide prevention were considered for inclusion. Suicide prevention interventions in this review included, but were not limited to, suicide risk assessment, safety planning intervention and lethal means restriction [7, 8]. Lastly, this review considered all ICT-based interventions that targeted patients of any age.

#### Context

All hospitals or clinical settings were considered for inclusion. For this review, a clinical setting was defined as any context where clinician-patient interactions occurred in real-time. Therefore, who implemented the ICT-based intervention was part of the context. To be considered for inclusion, ICT-based interventions needed to be implemented and initiated in clinical settings. Therefore, this review excluded crisis services because they are first initiated by patients, often in a public context, which we assumed to have different implementation characteristics compared to ICTs initiated in clinical settings. Additionally, there has already been a systematic review investigating effectiveness of crisis lines [43]. Self-support tools that patients can freely download from app stores or tools that involved self-referrals after reading public advertisements were also excluded as these were being initiated in non-clinical settings. Further to this, studies focusing on the development of ICT(s) without implementation were excluded. See Table 1 for summary of eligibility criteria.

**Table 1:** Inclusion criteria

	Inclusion criteria
Population	All members of clinical care team • Licenced and regulated practitioners • Unregulated practitioners or clinical support teams such as peer support workers
Concept	• Information and Communication Technologies (ICTs): “A set of technologies resulting from the convergence of information technology and advanced multimedia and telecommunications techniques, which have enabled the emergence of more efficient means of communication, by improving processing, storage, distribution and exchange some information” [13] • Suicide-related thoughts and behaviours [40]: represent a spectrum of suicide-related ideation, communication, behaviours and attempts with having casual to persistent suicidal thoughts with actual, undetermined or no suicidal intent (e.g., NSSI). This review will consider ICT-based interventions for suicide prevention regarding any sub-category of suicide-related thoughts and behaviours • Suicide prevention interventions included but was not limited to the following list adapted from Wilson [7] and Zalsman [8] • Screening and assessment • Safety plan (e.g., identifying warning signs coping strategies, emergency contacts) • Lethal means restriction and counselling • Discharge or post-discharge follow up • Behaviour or cognitive therapies
Context	Clinical/hospital setting or context (i.e., clinician-patient interaction in real time)
Source	• Primary research studies of any design • Study protocols • Conference papers, reports from relevant health services organizations
Language	English

### Search strategy

We worked with a health sciences librarian to develop a comprehensive search strategy to find relevant scholarly literature in several bibliographic databases. This scoping review followed a three-step search strategy outlined in JBI methodology [31, 32]. First, a librarian developed and refined a draft strategy in Medline, then analyzed text words and index terms contained in titles and abstracts of relevant articles as well as the subject headings. Second, relevant text words and index terms from the selected articles were used to develop a full search strategy. Third, the search strategy comprised of all identified keywords and index terms was adapted for all included databases. This required iterative steps of revising and testing, and the final search strategies were peer-reviewed by a second research librarian using the Peer Review of Electronic Search Strategy (PRESS) guidelines [44]. A librarian ran the search in the following databases on August 17–20, 2021: MEDLINE (Ovid), Embase (Elsevier), CINAHL (EBSCO), PsycINFO (EBSCO), Web of Science, and Library, Information Science and Technology Abstracts (LISTA). The selection of the listed databases was informed by consultation with a librarian, and they provide full coverage of literature likely to provide information specific to ICTs in clinical settings. All final search strategies are presented in Additional File 1.

Godin’s targeted Google search method [45] was used to locate additional eligible sources. First, we conducted ten unique Google searches with combinations of keywords and then reviewed the first ten pages of each search results to identify international and national health services websites. Second, we hand-searched relevant websites identified in the first step to find eligible sources. These two steps were carried out in incognito’ mode, which limited the impact of previous search history on new results. This Google search was complementary to the database searches to identify additional sources of evidence that our search strategy might have missed.

### Types of sources

All research study designs were included (e.g., quantitative, qualitative, mixed methods). Although study protocols did not have empirical data, we included them to capture relevant details. Protocols tend to include details on interventions and implementation, such as intervention components, implementation plans, implementation blueprints, and discrete implementation strategies. Such information is useful characteristics to identify. Furthermore, by including protocols, we can reflect upcoming trends, such as the most used research designs in the upcoming years. Reference lists of relevant literature reviews, commentaries, and opinion papers were reviewed to identify additional primary studies that met our eligibility criteria. We also considered grey literature for inclusion, such as conference papers and reports from relevant health organizations. Sources had to be available in English, and no date parameters were applied.

### Study selection

All identified citations were uploaded into Covidence [46] and duplicates were automatically removed by Covidence. Two reviewers (HDS, LS) independently screened titles and abstracts against the eligibility criteria. Next, relevant full-text articles were retrieved into Covidence [46], and the primary (HDS) and secondary reviewers (KD, LS) independently assessed them in detail against the eligibility criteria. Reasons for exclusion were recorded at the full-text screening phase and reported in the PRISMA flow diagram. Any discrepancies between the reviewers (HDS and LS or HDS and KD) at each stage were resolved either through discussion or by a third reviewer (KD or LS). Scoping reviews generally do not require methodological assessment [32], thus critical appraisal was not conducted.

### Data extraction

We developed an extraction tool in Covidence to capture characteristics of the paper, setting, participating clinicians, implementation strategies, descriptions of ICT-based intervention(s), patient population, barriers and facilitators to implementing ICTs, and reported measures and outcomes. Three reviewers (HDS, KD, LS) first pilot-tested the extraction tool on three studies to identify any discrepancies or inconsistencies prior to data extraction. Each person extracted the same three studies independently using the extraction tool. We initially proposed to pilot-test the extraction tool on five studies. However, after testing on three studies for calibration exercise, the team agreed that all relevant data were captured, so we decided to start independent extraction without testing on two more studies. Minor changes to the original extraction tool were made, such as extracting the reported use of theories, models, or frameworks. The primary (HDS) and secondary reviewers (KD, LS) independently extracted data using Covidence [46]. Any conflicts in data extraction were resolved either through discussion between the two reviewers (HDS and LS or HDS and KD) or by a third reviewer (KD or LS). Final version of the data extraction tool is included in Additional File 2.

### Data analysis

Following data extraction, we characterized extracted data using frameworks, typology, and taxonomy. First, identified ICT-based interventions for suicide prevention were categorized using a typology for e-Mental Health created by the Mental Health Commission of Canada (MHCC) [47]. Intervention descriptions were then characterized based on the suicide prevention interventions category adapted from Wilson [7] and Zalsman [8], and the WHO’s three levels of suicide prevention [1].

Second, we performed directed content analysis [48] using the Behaviour Change Wheel (BCW) [49, 50] and the Theoretical Domains Framework (TDF) [51] to map clinician-reported barriers and facilitators to implementing ICT-based interventions. They are comprehensive, evidence-based behaviour frameworks that capture internal and external influences of behaviour change. The Capability, Opportunity and Motivation – Behaviour (COM-B) model within the BCW explains behaviours by describing interactions between one’s capability, opportunity and motivation [49]. TDF is a 14-domain behavioural framework that expands the COM-B [51], so when used together, TDF allows for granularity of behaviour analysis [52]. Furthermore, benefits of using BCW and TDF for assessing implementation barriers and facilitators have been previously documented across healthcare disciplines [53–55]. Narrative descriptions of reported barriers and facilitators were mapped onto the COM-B and TDF.

Third, this review categorized reported measures and outcomes of ICT-based interventions for suicide prevention. Outcomes were categorized as either implementation outcomes or impact outcomes of the ICTs. Implementation outcomes were further categorized using Proctor’s Implementation Outcomes Framework: (1) Acceptability, (2) Adoption, (3) Appropriateness, (4) Feasibility, (5) Fidelity, (6) Implementation cost, (7) Penetration, and (8) Sustainability [56]. Impact outcomes or intervention outcomes were categorized into three levels: (1) Patient, (2) Health care provider (i.e., clinician), (3) Health system. Patient level impact was further categorized into patient-reported outcomes (PRO) [57], patient-reported experience (PRE) [58], and patient health outcomes (e.g., mortality) [59]. PRO comes from patients and often records patients’ view of their health status and condition [57]. Patients’ views of their own health can provide insight into the impact of an intervention [58]. In contrast to PRO, PRE measures patients’ perceptions and experiences of receiving care, providing insight into the quality of care during the intervention and the process of care [58]. Health care provider level outcomes include conceptual knowledge use (i.e., proximal practice change), instrumental knowledge use (i.e., observable practice change) [60], and other provider-reported experiences. Examples of conceptual knowledge use include levels of knowledge, and examples of instrumental knowledge use include rates of completed assessments [60]. Lastly, system level outcomes include resource utilization and economic outcomes such as cost effectiveness, and readmission rates [59]. Additional File 3 provides the full coding strategy with operationalized definitions.

The coding strategy was pilot tested on three studies by the primary reviewer (HDS), who has experience in qualitative research. Then second reviewers (LS, KD) who also have qualitative research experience reviewed the coded data generated by HDS to identify discrepancies and ensure consistency in coding. LS reviewed the coded data for barriers and facilitators and KD reviewed the categorized outcomes. LS and KD each reviewed half of the coded data for the characteristics of ICT-based interventions for suicide prevention. No changes were made to the coding strategy after pilot testing, and the primary reviewer (HDS) coded the rest of the data. Then the second reviewers (KD, LS) reviewed all coded data to verify HDS’s work. Any disagreements between the reviewers were resolved through discussion.

### Data summarizing and reporting results

We charted the data in a tabular form that aligns with the review questions and aim. We also produced descriptive numerical summaries of the quantitative data (i.e., frequency counts) and graphical figures. We then provided narrative summaries to accompany these presentations and addressed the review questions and aim.

## Results

**Fig. 1:**
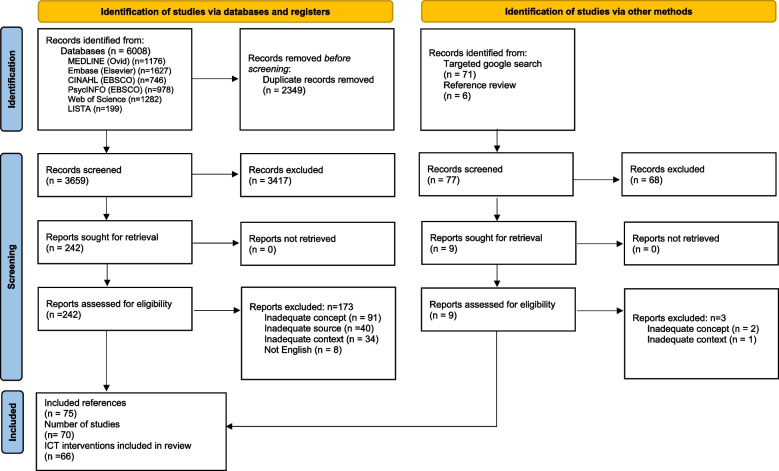
PRISMA flow chart

Our database searches resulted in 6,008 citations. After duplicate removal, 3,659 citations remained for assessment against the eligibility criteria. After screening titles and abstracts, 242 citations remained for full-text review, and we identified an additional 6 relevant papers through Google searches and reviewing references of relevant reviews. This review included a total of 75 citations, describing 70 studies and 66 ICT-based interventions. See Fig. 1 for the PRISMA flow chart which includes the reasons for excluding full-text articles.

### Characteristics of included studies

Of the 70 papers, there were 52 research studies and 18 study protocols. There were five protocols of completed studies (i.e., protocol-study dyads) [61–65]. Seventy studies were a mix of experimental design (*n* = 22) [66–87], observational design (*n* = 12) [88–99], qualitative design (*n* = 3) [100–102], case study (*n* = 1) [103], quality improvement report (*n* = 1) [104], and feasibility/pilot trial (*n* = 31) [105–135] that served as a precursor to a larger study. These 70 studies originated from USA (*n* = 32) [69, 71, 73, 75, 76, 78, 81, 83, 88–90, 94–96, 98, 103, 104, 106, 109, 112–116, 118, 120–122, 124, 125, 132, 134], France (*n* = 8) [68, 72, 77, 86, 87, 91, 92, 105], UK (*n* = 8) [84, 97, 101, 107, 108, 131, 133, 135], Australia (*n* = 5) [67, 85, 117, 126, 128], Denmark (*n* = 5) [66, 82, 100, 127, 130], Canada (*n* = 4) [74, 93, 102, 111], South Korea (*n* = 2) [99, 119], Netherlands (*n* = 1) [129], Iran (*n* = 1) [80], Sri Lanka (*n* = 1) [79], Japan (*n* = 1) [123], Spain (*n* = 1) [70], and Portugal (*n* = 1) [110]. Studies took place most commonly in out-patient clinical settings (*n* = 43) [66, 67, 69–71, 73, 75, 76, 82–88, 90–92, 94, 96, 99–103, 105–107, 109–111, 114, 115, 117, 118, 123, 125, 127, 129–132, 134], such as emergency departments and clinics, then in-patient clinical settings (*n* = 14) [77–80, 93, 97, 98, 108, 113, 120–122, 128, 135], such as in-patient psychiatric units, and a mixture of both (*n* = 11) [68, 72, 74, 81, 89, 95, 104, 116, 119, 124, 133]. The remaining two studies were conducted in mental health hospitals but did not report specific clinical setting characteristics [112, 126]. Examples of involved clinicians included psychiatrists, nurses, physicians, social workers, and behaviour health clinicians, such as psychologists. Lastly, there was a lack of reported theories, models, or frameworks (TMFs) guiding research. Seven studies explicitly reported TMFs guiding their research [116, 118, 119, 124, 129, 131, 135], including the User-Centered Design Principles, Proctor’s Implementation Outcomes Framework, Theory of Planned Behaviour, Interpersonal Psychological Theory of Suicide, Integrated Motivational-Volitional model of suicidal behaviour, Medical Research Council, Process evaluation framework for analysis, and Normalisation Process Theory. Table 2 summarizes overall characteristics of included papers.

**Table 2:** Characteristics of included studies

Author, Year	Country of origin	Study design	Research aim/objectives/ questions	Patient population	Clinical setting and type	Clinician characteristics
Andreasson et al., 2017 [66] (Protocol)	Denmark	Experimental	-Investigate if a safety planning tool delivered as an app, compared to a safety plan delivered by paper, can reduce suicide ideation after 12 months of intervention in patients referred to Suicide Prevention Clinics	Patients from Suicide Prevention Clinics	Seven Suicide Prevention Clinics and their satellite sites. Patients are typically referred to the clinics from somatic and psychiatric EDs after a self-harm episode Out-patient	Clinicians working at the Suicide Prevention Clinics
Bailey et al., 2020 [67]	Australia	Experimental	-Evaluate the safety, feasibility, and acceptability of a MOST intervention (“Affinity”) among a sample of young people who were receiving treatment for major depressive disorder and had also experienced past-four-week suicidal ideation. -Explore changes in cognitive and interpersonal targets of the Affinity intervention, as well as changes in self-reported depression and suicidal ideation	Patients with suicidal ideation within the past four weeks	The Youth Mood Clinic (YMC), a tertiary-level outpatient mental health service that is part of Orygen, a state government-funded youth mental health service in Melbourne, Australia. YMC specialises in the treatment of young people with complex depression Out-patient	Youth mental health clinicians from the youth mood clinic and treating clinicians and Affinity staff
Berrouiguet and Alavi et al., 2014 [61] (Protocol) HUGOPSYNetwork et al., 2018 [68]	France	Experimental *Only reported descriptive results on selected cases	-Determine whether the receipt of a text message sent regularly over a six-month period can reduce suicidal and self-harming behaviour among suicide attempters -Identify cases of patients recruited in the SIAM study that may demonstrate the capability of a mobile-based brief contact intervention for triggering patient -Initiated contact with a crisis support team at various time points throughout the mobile-based follow-up period	Patients who attempted suicide	Psychiatric EDs and psychiatric units. Public funded specialist mental health services for adults (Brest, Rennes, Nantes, Lille, Angers, Tours, Vannes) Mixture	Psychiatrist, general physician
Berrouiguet and Gravey et al., 2014 [105]	France	Pilot/Feasibility trial	-Assess the technical feasibility of an automated and tailored text messaging tool in a sample of suicidal patients. -Assess the patient's acceptability of such intervention through a phone interview	Patients who attempted suicide	Psychiatric ED Out-patient	Psychiatrist, general physician
Betz et al., 2020 [106]	United States	Pilot/Feasibility trial	-Test the feasibility and acceptability of Lock to Live (L2L) among suicidal adults in EDs	Patients with identified suicide risk	4 large EDs in Colorado: A tertiary care academic center, an urban safety net hospital, and a regional medical center with 2 EDs in a geographic region with firearm ownership rates that are higher than state averages. All EDs had 24/7 coverage by behavioral health specialists Out-patient	Not reported
Brand and Hawton, 2021[107]	United Kingdom	Pilot/Feasibility trial	-Ascertain the usefulness for patients and clinicians of a digital self-monitoring system alongside outpatient follow-up after patients had presented to a general hospital with self-harm	Patients with self-harm	A large general hospital in Oxford, England. The Emergency Department Psychiatric Service (EDPS) is based in a large general teaching hospital and offers psychosocial assessment to anyone aged over 13 years who presents to a hospital ED following an episode of self-harm or any other mental health issue Out-patient	Five nurses in the EDPS team
Bruen et al., 2020 [108]	United Kingdom	Pilot/Feasibility trial	-Report the practicalities and acceptability of setting up and trialling digital technologies within an inpatient mental health setting in the United Kingdom and to highlight the implications of these for future studies	Services users from acute adult mental health wards	6 National Health Service acute mental health wards in Northwest United Kingdom In-patient	Not reported
Bush et al., 2015 [109]	United States	Pilot/Feasibility trial	Research questions:-Can a smartphone app be developed that contains the essential elements of a hope box and associated elements of CT/DBT in a package acceptable to and usable by military service members and veterans? -Is the VHB app as usable, acceptable, convenient, and ostensibly useful as a conventional hope box to a clinical sample of service veterans at high risk of self-harm and suicide and their providers?	High-risk of self-harm veterans who either had borderline personality disorder, bipolar disorder, treatment refractory depression, or PTSD	Large, regional Veteran Administration (VA) behavioral health clinic Out-patient	6 Clinical social workers and one clinical psychologist, with a mean of 7.9 years (range 1–16 years) in practice
Bush et al., 2017 [69]	United States	Experimental	-Assess the primary impact of Virtual Hope Box (VHB) on stress coping skills over 12 weeks, the secondary impact of VHB on suicidal ideation and reasons for living, the use of VHB for addressing emotional dis-equilibrium away from the clinic, and the patient experience of VHB through objective usage patterns and self-reported usability and perceived benefits	Veterans who currently expressing suicidal ideation or had expressed suicidal ideation within the three months before recruitment	13 Treatment programs within Veteran Mental Health Care (Outpatient—Veterans Affairs Portland Health Care System) Out-patient	Behavioral health clinicians
Buus et al., 2020 [100]	Denmark	Qualitative	-Explore different stakeholder perspectives on the MYPLAN app for suicide prevention safety planning	Young and adult users with variations in psychosocial problems	Clinics that offer short-term, specialized psychosocial therapy to patients at risk of suicide Out-patient	Clinicians with median age of 46 (range: 37–60). Female: *n* = 9 Male: *n* = 1
Cassola et al., 2017 [110] (Protocol)	Portugal	Pilot/Feasibility trial	-Understand the health professional’s satisfaction on the use of the platform for depression and suicidality	Patients with depression and suicidality	Primary health care setting Out-patient	18 Primary care health professionals
Cebrià 2013 [70]	Spain	Experimental	-Determine the effectiveness of this specific telephone management on patients	Patients who attempted suicide	Emergency room of Corporacio ´Sanitaria Parc Taulı ´(CSPT) that covers an area of 400,000 inhabitants and provides urgent medical attention for all suicidal behaviours Out-patient	Nurse
Chen et al., 2010 [111]	Canada	Pilot/Feasibility trial	-Determine whether a mobile telephone message intervention would be acceptable to suicide attempters -Explore the operational procedures of this intervention to help determine the appropriate content of supportive messages -Test the feasibility of cell-phone message interventions	Patients who attempted suicide	EDs of two general hospitals, Tongji Hospital and Union Hospital, in Wuhan, China Out-patient	Nurses and psychologists
Chen et al., 2018 [88]	United States	Observational	-Describe usage of specific app subcomponents and to determine if specific demographic and clinical characteristics were associated with higher or lower overall use of the VHB -Explore the association between usage of the VHB and psychosocial outcomes	Patients with recent or ongoing suicidal ideation and were engaged in active mental health treatment	13 Clinical programs at a large, north-western Veterans Health Administration hospital Out-patient	Not reported
Comtois et al., 2019 [71]	United States	Experimental	-Test the effectiveness of augmenting standard military health care with Caring Contacts delivered via text message to reduce suicidal thoughts and behaviors over 12 months	Patients with suicidal ideation or suicide attempt	3 Military installations: an Army base in the southern United States, a Marine Corps base and air stations in the southern United States, and a Marine Corps base in the western United States Out-patient	Licensed masters level mental health clinicians, who were called continuity clinicians and credentialed as behavioural health clinicians
Czyz et al., 2021 [113]	United States	Pilot/Feasibility trial	-Investigate the feasibility and acceptability of SMART study procedures, including the sequencing of intervention components	Patients with suicidal ideation or suicide attempt	In-patient psychiatric unit In-patient	A total of 3 masters level training in psychology or social work (Counselors)
Czyz et al., 2020 [112]	United States	Pilot/Feasibility trial	-Describe the process of development and report on the feasibility and acceptability of the text-based intervention as a continuity of care strategy promoting coping and safety plan use following discharge	Patients with suicidal ideation or suicide attempt	Adolescent mental health hospital Undetermined	Not reported
Davis et al., 2021[89]	United States	Observational	-Describe levels of adolescent suicide risk detected via depression screening in a large primary care network-Understand fidelity to the system’s suicide risk assessment procedures Examine follow-up for adolescents at-risk for suicidality in the year after risk was detected	Patients with suicidal ideation or suicide attempt	A large pediatric healthcare facility Mixture	Not reported
Depp et al., 2021[114] (Protocol)	United States	Pilot/Feasibility trial	-Refine intervention content and safety protocol with input from community stakeholders. -Evaluate feasibility, engagement, impact, and preliminary comparison of START with Mobile Augmentation versus START alone	Patients with DSM-5 of bipolar disorder, schizoaffective disorder, or schizophrenia and having suicidal ideation	Public mental health system (Walk-in or same-day clinics) in San Diego, California Out-patient	A triage provider (typically a social worker)
Dimeff et al., 2020 [116]	United States	Pilot/Feasibility trial TMF: User-centered design principles	-Design, develop, and evaluate the feasibility of “Dr. Dave” and the Virtual CAMS system, including electronic “Caring Contacts,” for suicidal patients in EDs, as well as a provider-facing clinical decision support tool to aid in discharge disposition to reduce unnecessary hospitalization	Patients with identified acute suicide risk	EDs and 3 private outpatient specialty clinics Mixture	21 Medical providers
Dimeff et al., 2021 [115]	United States	Pilot/Feasibility trial	-Examine the feasibility, acceptability, and effectiveness of Jaspr Health for adults who were acutely suicidal in the ED	Patients with suicide attempt and/or a lifetime history of engaging in non-suicidal self-injurious behaviors	2 EDs from large health systems in Midwest US. Each ED offered 24/7 psychiatric care offered by behavioral health providers Out-patient	Behavioural health providers, masters level social workers and physician, psychiatrist and psychiatric nurse practitioner
Duhem et al., 2018 [72] (Protocol)	France	Experimental	-Implement an adaptive recontact system that smoothly and effectively combines surveillance and different types of Brief Contact Interventions that fit each patient’s specific needs.-Optimise the care management of patients discharged from the hospital after a suicide attempt by providing health stakeholders with standardised tools, effective skills and specialised literacy -Offer professionals involved in the follow-up of suicide attempters a readily available alert network to improve their coordination and reactivity in case of new suicidal crises	Patients who attempted suicide	A total of 28 Centres in Nord–Pas-de-Calais region: EDs, psychiatry crisis centres, psychiatry departments, and private clinics Mixture	Coordination team, and a call team consisting of 3 psychologists and 3 psychiatric nurses
Etter et al., 2018 [90]	United States	Observational	-Assess the use of a computerized clinical decision support system (CDSS) to screen adolescents for suicide risk, deliver follow-up recommendations to the provider, and document actual provider follow-up actions in a primary care setting	Patients who presented to pediatric primary care clinic for an annual or sick visit	Federally qualified health center clinics that utilize Child Health Improvement through Computer Automation (CHICA) and are part of an urban, Midwest County hospital system (Eskenazi Health) Out-patient	Physicians were primarily trained in pediatrics, family medicine, and combined internal medicine and pediatrics, with some having completed subspecialty fellowship training in adolescent medicine
Fossi Djembi et al., 2020 [91]	France	Observational	-Test the hypothesis of a correlation between the decrease of SA rate and the amount of coverage of VigilanS	Patients who attempted suicide	21 hospitals (EDs) in the Nord-Pas-de-Calais region Out-patient	Mental health care professionals specially trained in suicidal crisis management
Fossi et al., 2021[92]	France	Observational	-Describe the characteristics of the patients, to estimate the mean time between suicidal iterations, and to identify the profiles of patients who had a suicide reattempt compared to other patients	Patients who attempted suicide	Emergency Department in regional France Out-patient	Not reported
Goodman et al., 2020 [73] (Protocol)	United States	Experimental	-Examine if Veterans who are at high-risk for suicide will benefit from the novel group intervention, PLF, compared to Veterans who receive TAU (e.g., individual safety planning)	Patients with suicidal ideation or suicide attempt	Multiple sites of Veterans Health Administration (VHA) in New York and Philadelphia Out-patient	2 Therapists
Gregory et al., 2017 [93]	Canada	Observational	-Examine whether or not we could effectively integrate smartphone-based safety planning into the discharge process on a child and adolescent inpatient psychiatry unit	Patients discharged from the child and adolescent psychiatry inpatient unit	The child and adolescent psychiatry inpatient unit at London (Ontario) Health Sciences Centre In-patient	Unit staff (either nurses or child and youth counsellors)
Grist et al., 2018 [101]	United Kingdom	Qualitative	-Explore the acceptability, usability, and safety of BlueIce with young people aged 12–17 years who are self-harming and attending child and adolescent mental health services (CAMHS)	Patients with self-harm	CAMHS provided by Oxford Health NHS Foundation Trust. The Trust provides mental healthcare for children and young people in Buckinghamshire, Oxfordshire, Swindon, Wiltshire, and Bath and North-East Somerset Out-patient	37 clinicians
Gros et al., 2011 [103]	United States	Case study	-The case report concerns a US veteran of the Afghanistan war with PTSD, who developed severe suicidal ideation	One patient (case report) -45yrs old -PTSD -Suicidal ideation-African American -Male-Veteran -Lived in trailer with two adult children -Divorced	Hospital in South-east US Out-patient	2 Therapists
Hatcher et al., 2020 [74] (Protocol)	Canada	Experimental	-Evaluate the relationship between the amount of smartphone-assisted problem-solving therapy (PST) and suicidal ideas in men over a 1-year period	Patients with self-harm	10 Sites from the department of psychiatry and department of emergency medicine in Ontario, Canada Mixture	Not reported
Hetrick et al., 2017 [117]	Australia	Pilot/Feasibility trial	Research Questions:-Whether the online depression and suicidal ideation monitoring tool was feasible in terms of improving monitoring, -How acceptable and useful the tool was for clinicians and clients, and -Whether a refined (shorter) tool could be implemented	Patients with depressive symptoms or a depressive disorder	One primary, two enhanced primary care, and one tertiary care setting in Victoria. In the primary care setting within a routine general practice, mental health care was provided to clients of all ages. The tertiary care setting was a public mental health service (Orygen Youth Health) for young people aged 15–24 years Out-patient	Clinicians from a range of backgrounds including clinical psychologists and other allied health professionals
Hill et al., 2020 [118]	United States	Pilot/Feasibility trial TMF: Proctor ‘s Implementation Outcomes Framework	-Evaluate whether use of the Safety Planning Assistant resulted in high quality, completed safety plans in a timely manner and to evaluate participant satisfaction with the Safety Planning Assistant and participant completion of the intervention modules	Patients with identified suicide risk	Pediatric hospital in major metropolitan area Out-patient	Social worker
Jeong et al., 2020 [119]	Korea	Pilot/Feasibility trial TMF: Theory of Planned Behaviour	-Develop and evaluate a safety plan mobile app based on the TPB for adolescent suicide attempt survivors (study 1) -Evaluate its effectiveness for target users (study 2)	Patients who attempted suicide	A mental health promotion center in Seoul Mixture	Study 1: 6 healthcare professionals who all worked in an emergency or psychiatry department. They all were involved in treatment, nursing, or consultation of adolescent suicide attempt survivors
Jerant et al., 2020 [75]	United States	Experimental	-Examine the effect of Men and Providers Preventing Suicide (MAPS) on discussion of suicide during primary care clinician visits by middle-aged men with recent active suicidal thoughts. -Explore moderation of the program’s effects by the presence of suicide preparatory behaviours, a risk marker for suicide	Men who were assigned to the panel primary care clinician	Primary care offices in Sacramento (California) area Out-patient	32 Primary care clinicians: 21 (65%) were family physicians and 11 (35%) were general internists; they had practiced on aver-age for 8 years (range 1–22); their mean age was 44 (range 29–61); 21 (65%) were female; 19 (59%) were non-Hispanic White, 7 (22%) non-Hispanic Other race, and 5 (26%) Hispanic
Kasckow et al., 2015 [76]	United States	Experimental	-Test the hypothesis that use of the telehealth system would result in a greater reduction in both suicidal ideation and depressive symptoms on standardized measures following discharge from an inpatient service, relative to a group that received only Usual Care. -Assess feasibility of telehealth monitoring for suicidal behavior in this population	Veterans with recent suicidal ideation or a recent suicide attempt and a diagnosis of schizophrenia or schizoaffective disorder	Veterans Affairs in Pittsburgh Out-patient	Nurses
Kasckow et al., 2016 [120]	United States	Pilot/Feasibility trial	-Test the feasibility of the telehealth monitoring intervention for suicidal behavior in this population of Veterans with schizophrenia or schizoaffective dis-order-Assess with a random assignment trial, whether augmentation of intensive care monitoring (ICM) with our intervention would result in a significant reduction in suicidal ideation relative to a group that received only ICM	Admitted patients with a diagnosis of schizophrenia/ schizoaffective disorder and recent suicidal ideation	Inpatient psychiatric unit, Veterans Affairs In-patient	Nurses
Kennard et al., 2018 [121]	United States	Pilot/Feasibility trial	-To report on a pilot study of an inpatient intervention for suicidal adolescents, As Safe as Possible [ASAP], supported by a smartphone app [BRITE] to reduce post-discharge suicide attempts	Patients with suicidal ideation, intent and/or a recent suicide attempt	Psychiatric inpatient units at two academic medical centers In-patient	A total of 5 therapist who had at least master’s level training in psychology/counseling or were enrolled in a clinical psychology doctoral program
Kleiman et al., 2019 [122]	United States	Pilot/Feasibility trial	-Examine whether participants would wear the monitor the majority of each day over the course of multiple days.-Investigate whether participants would interact with the monitor (i.e., use the self-initiated button press). -Investigate what participants liked (or disliked) about wearing the monitor	Admitted patients with severe suicidal ideation, suicide attempt, or non-suicidal self-injury	Psychiatric inpatient units at two academic medical centers in New Jersey In-patient	Not reported
Kodama et al., 2016 [123]	Japan	Pilot/Feasibility trial	-Identify whether suicide interventions sent via mobile phone text messaging technologies is feasible in changing help-seeking and self-harming behaviours	Patients with a mental disorder and suicidal ideation	University hospital, a psychiatric hospital in Hyogo Prefecture, 3 medical center hospitals in Kobe City, a private psychiatric hospital, and 3 psychiatric clinics in Kobe City Out-patient	Psychiatrists
Kolva et al., 2020 [94]	United States	Observational	-Discuss an approach to preserve patient safety while optimizing delivery of an online survey of suicidality in cancer survivors seeking psychological care	Patients from outpatient psycho‐oncology comprehensive cancer center	Outpatient psycho-oncology clinic Out-patient	Psycho‐oncology provider
Kroll et al., 2020 [95]	United States	Observational	-Determine whether continuous virtual monitoring, an intervention that facilitates patient observation through video technology, can be used to monitor suicide risk in the general hospital and ED	Patient who received a psychiatric consultation and required suicide precautions	An academic tertiary adult hospital (Boston, Massachusetts) with 793 licensed inpatient beds Mixture	Nurses and psychiatrists and psychiatry trainees
Lawrence et al., 2010 [96]	United States	Observational	-Implement routine self-administered computerized screening for suicidal ideation linked to automated activation of a response team in two high volume, urban HIV clinics-Identify factors associated with self-reported suicidal ideation as determined by computerized screening in a contemporary sample of HIV-infected individuals	Patients with HIV	Two geographically distinct academic HIV primary care clinics: University of Alabama at Birmingham HIV/AIDS Clinic Cohort and the University of Washington (UW) Harborview Medical Center HIV Clinic Out-patient	4 Licensed mental health professional and social worker supervisor and physician
Levine et al., 1989 [97]	United Kingdom	Observational	-Assess the incidence of depressive symptoms in patients admitted following deliberate self-harm using a self-rating modification of Hamilton Rating Scale for Depression delivered by delivered by computer-Compare initial clinical assessment with outcome in those patients who went on to commit suicide	Patients who attempted suicide	District General Hospital In-patient	Psychiatrist
Ligier et al., 2016 [77] (Protocol)	France	Experimental	-Determine whether a short message service in addition to usual care can be used to: keep in touch with adolescent suicide attempters to reduce the delay in recurrence of a suicide attempt, and to improve the evolution of 1) their social network, 2) depression and 3) health-related quality of life	Patients who attempted suicide	Pediatric and adolescent psychiatry unit at hospitals in eastern France: CHU Besançon, CHU Dijon, CHR Metz-Thionville, CHU Nancy, CHU Reims, and CHU Strasbourg In-patient	Physicians
Luxton et al., 2012 [124]	United States	Pilot/Feasibility trial TMF: Interpersonal Psychological Theory of Suicide	-Evaluate the program to determine how to best tailor the caring letter intervention to the military setting-Explore preliminary group differences related to psychiatric rehospitalizations-Compare the use of handwritten letters versus e-mail correspondence -Gather data to inform best practices that will assist the development of a multisite RCT	Retirees, veterans, and dependent family members admitted in in-patient psychiatric units	Veterans Hospital Mixture	Inpatient psychiatry treatment team consisting of psychiatric nurse
Luxton et al., 2014 [62] (Protocol) Luxton et al., 2020 [78]	United States	Experimental	-Determine whether the intervention is efficacious in preventing suicide behaviours among U.S. service members and veterans	Veterans who are currently admitted to psychiatric inpatient units	Inpatient psychiatry units: Madigan Army Medical Center, Tripler Army Medical Center, Landstuhl Regional Medical Center, Navy Medical Center San Diego, Veterans Affairs Palo Alto, and Veterans Affairs Western New York In-patient	Not reported
Mackie et al., 2017 [102]	Canada	Qualitative	-Inform the production of a treatment manual for a larger cluster randomised trial of a smartphone-assisted therapy for men who present to hospital after intentional self-harm. -Describe the experience of receiving and delivering a novel blended therapy combining a customised smartphone application with problem solving therapy (PST) for this population	Patients with self-harm	Emergency department in a major Canadian urban centre (The Ottawa Hospital) Out-patient	Psychiatrists, therapists
Madan et al., 2015 [98]	United States	Observational	-Describe integration of an electronic suicide risk alert system to improve assessment of psychiatric, high-risk patients-Provide support of using aggregate data over time to inform administrative and clinical decision-making related to changes in the treatment delivery system	Patients admitted at the specialty psychiatric hospital	Specialty psychiatric hospital (Menninger Clinic, Huston Texas): a 120-bed facility that specializes in the treatment of individuals with serious mental illness In-patient	Nurses
Marasinghe et al., 2012 [79]	Sri Lanka	Experimental	-Test whether a Brief Mobile Treatment (BMT) intervention can improve outcomes relative to usual care among suicide attempters	Admitted patients with self-harm	Colombo South Teaching Hospital in Kalubowila, Sri Lanka In-patient	Not reported
McManama O'Brien et al., 2017 [125]	United States	Pilot/Feasibility trial	-Test the usability, feasibility, and acceptability of a web-based prototype of Crisis Care with 20 adolescents with a history of suicidal thoughts and their 20 parents	Patients from outpatient psychiatry department	Outpatient psychiatry department at a general pediatric hospital in Northeast US Out-patient	Not reported
Melvin et al., 2019 [126]	Australia	Pilot/Feasibility trial	-Examine the feasibility and effectiveness of a suicide prevention smartphone application	Patients from tertiary mental health service, and most of them had depressive disorder and suicide attempt	Tertiary mental health service in Melbourne, Australia Undetermined	Not reported
Morthorst et al., 2021 [127] (Protocol)	Denmark	Pilot/Feasibility trial	-Assess the feasibility of methods, procedures, and safety of internet-based Emotion regulation individual therapy (ERITA) in a Danish context	Patients with non-suicidal self-injury (NSSI)	Outpatient clinics in The Child and Adolescent Mental Health Services in capital region on Denmark Out-patient	Psychologists and nurses with experience within clinical child and adolescent psychiatry and with psychotherapy and special knowledge about NSSI
Mousavi et al., 2014 [80]	Iran	Experimental	-Evaluate the efficacy of telephone follow up on reduction of suicidal reattempt and their relationship with demographic characteristics of patients	Patients who attempted suicide	Intoxication emergency services, Noor Hospital, Isfahan In-patient	Psychiatry last-year resident
Muscara et al., 2020 [128]	Australia	Pilot/Feasibility trial	-Assess the feasibility and acceptability of a combination of smartphone apps to deliver a digitized safety plan, BeyondNow, and personalized management strategies, BlueIce, with adolescents discharged from a mental health inpatient ward following self-harm, suicidal ideation and/or behavior.-Explore whether any changes in suicide resilience and self-harming behaviors were able to be detected six weeks following discharge	Admitted patients with suicide attempt	Inpatient mental health ward (Banksia) at the Royal Children's Hospital in Melbourne, Australia In-patient	Not reported
Nuij et al., 2018 [129] (Protocol)	Netherlands	Pilot/Feasibility trial TMF: Integrated Motivational-Volitional (IMV) model of suicidal behaviour	-Evaluate the feasibility of mobile safety planning and daily mobile self-monitoring in routine care treatment for suicidal patients, and to conduct fundamental research on suicidal processes	Patient with main diagnosis of major depressive disorder or dysthymia and current suicidal ideation	3 Mental health organizations Out-patient	Not reported
O'Keefe et al., 2019 [81] (Protocol)	United States	Experimental	Evaluate which brief interventions, alone or in combination, have the greater effect on suicide ideation (primary outcome) and resilience (secondary outcome) among American Indian youth ages 10–24 ascertained for suicide-related behaviours by the tribal surveillance system	American Indian/ Alaska Indian youth with suicide ideation, suicide attempt or binge substance use with suicide ideation	WMAT suicide surveillance system (locally known as “CelebratingLife”), mental health centres located in Fort Apache Indian Reservation in Eastern Arizona Mixture	Trained Apache Community mental health specialists
O'Toole et al., 2019 [82]	Denmark	Experimental	-Compare the effect between treatment as usual (TAU) with (TAU + APP) and without (TAU) the assistance of the mobile app on individuals referred to outpatient suicide prevention treatment	Patients with suicidal ideation or suicide attempt	A specialized outpatient suicide prevention clinic located at a psychiatric university hospital in Denmark. The clinic provides psychosocial therapy for people at risk of suicide, typically presenting with adjustment disorders and mild to moderate depression Out-patient	Therapists
O’Connor et al., 2019 [135] (Protocol)	United Kingdom	Pilot/Feasibility trial TMF: Medical Research Council, Process evaluation framework for analysis	-Determine whether a safety planning intervention (SPI) with follow-up telephone support (SAFETEL) is feasible and acceptable to patients admitted to UK hospitals following a suicide attempt.-Adapt/tailor an innovative SPI with follow-up tele-phone support for use within UK NHS hospital settings.-Investigate how participants engage with the intervention. -Assess feasibility and acceptability of the intervention.-Investigate trial recruitment, retention and other trial processes including data collection.-Explore the barriers and facilitators to intervention implementation.-Collect data on readmission to hospital following self-harm in the 6 months following the index suicide attempt to inform the sample size required for a full trial.-Further develop and test the logic model and theoretical basis of the intervention -Assess whether an effectiveness trial is warranted	Admitted patients with suicide attempt	4 National Health Service hospitals across two health boards in Scotland In-patient	The Liaison Psychiatry team
Olsen et al., 2021 [130] (Protocol)	Denmark	Pilot/Feasibility trial	-Assess the feasibility and safety of Internet-based ERITA as an add-on to treatment as usual in 13–17-year-old patients with NSSI referred to the Child and Adolescent Mental Health Service	Patients with non-suicidal self-injury	Child and Adolescent Mental Health Services in capital Region of Denmark Out-patient	Therapists
Owens and Charles, 2016 [131]	United Kingdom	Pilot/Feasibility trial TMF: Normalisation Process Theory	-Test and refine the intervention in situ, before proceeding to a full trial Research question: -Can TeenTEXT be administered by CAMHS clinicians within the context of everyday clinical practice?	Patients with self-harm	Three Child and Adolescent Mental Health Services (CAMHS) teams in South West England Out-patient	CAMHS Clinicians
Canady 2018 [104]	United States	Other: Quality improvement	-Describe steps in developing and implementing this quality improvement program	Patients in ED and in-patient units	ED and inpatient units, Dallas-based hospital Mixture	Nurses
Pickett et al., 2021 [132]	United States	Pilot/Feasibility trial	-Determine the feasibility of implementing a self-administered tablet-based suicide screening questionnaire in an ED	Patients in a children's hospital ED	ED from children's hospital with an annual census of 70 000 patient visits Out-patient	Nurses and nursing assistants
Sayal et al., 2019 [133]	United Kingdom	Pilot/Feasibility trial	-Determine the acceptability and feasibility of carrying out an RCT of remotely delivered (video-calling or mobile phone) problem-solving cognitive behaviour therapy (PSCBT) plus treatment as usual (TAU) versus TAU in adolescents and young adults with depression who self-harm	Patients with self-harm	Adult or child and adolescent mental health services that assess people in emergency rooms or hospital wards following a self-harm presentation, adult or child and adolescent community mental health services that see people with depression and self-harm, a third sector organization providing interventions and support to people who have self-harmed Mixture	Cognitive behaviour therapist
Seong et al., 2021 [99]	Korea	Observational	-Investigate the effects of Mobile Messenger Counseling on the post-discharge case management results for patients with suicide attempts or self-harm	Patients with self-harm or suicide attempt	Regional ED center that operates through the use of a dedicated medical team for patients who have attempted self-harm or suicide Out-patient	Physicians, psychiatrists, and social workers
Simon et al., 2016 [63] (Protocol) Simon et al., 2022 [83]	United States	Experimental	-Compare 2 low-intensity outreach programs with usual care for prevention of suicidal behavior among outpatients who report recent frequent suicidal thoughts	Patients from an out-patient mental health or general medical visit who self-harm	3 Mental health outpatient care in Colorado. These health systems provide general medical and mental health specialty care as well as insurance coverage to defined member/patient populations Out-patient	Care managers, Skills coach (Master’s-prepared mental health professional)
Stallard et al., 2016 [64] (Protocol) Stallard et al., 2018 [84]	United Kingdom	Experimental	-Undertake a preliminary evaluation of a smartphone app (BlueIce), co-produced with young people and designed to help young people manage distress and urges to self-harm. -Assess the acceptability, safety, and use of BlueIce and to explore the effects on the primary outcome of self-harm and the secondary outcomes of psychological functioning	Patients with self-harm	Specialist child and adolescent mental health services provided by Oxford Health NHS Foundation Trust. The Trust serves a wide geographical area that includes Bath and North East Somerset, Buckinghamshire, Oxfordshire, Swindon, and Wiltshire Out-patient	A total of 37 clinicians: Child psychiatrists, clinical psychologists, family therapists, child psychotherapists, occupational therapists, and community psychiatric nurses
Stevens et al., 2019 [85] (Protocol)	Australia	Experimental	-Investigate whether Treatment As Usual (TAU) aftercare for DSH patients plus supportive SMS text messages delivered over 1-year reduce DSH re-presentations to hospital, compared to TAU alone	Patients with self-harm	Three public hospitals (EDs) in Western Sydney. Nepean, Blacktown and West-mead Hospitals (Australia): Toxicology Centers, Psychiatric Emergency Care Centers, and Mental Health Triage and Assessment Centers Out-patient	Psychiatrists, clinical nurse consultants, registered nurses, psychiatry registrars
Vaiva et al., 2006 [87]	France	Experimental	-Determine the effects over one year of contacting patients by telephone one month or three months after being discharged from an emergency department for deliberate self-poisoning compared with usual treatment	Patients who attempted suicide	13 EDs from north of France Out-patient	Psychiatrists with at least five years’ experience in managing suicidal crises
Vaiva et al., 2011 [65] (Protocol) Vaiva et al., 2018 [86]	France	Experimental	-Assess the effectiveness of a decision-making algorithm for suicide prevention (ALGOS) combining existing Brief Contact Interventions in reducing suicide reattempts in patients discharged after a suicide attempt	Patients who attempted suicide	23 EDs and psychiatry crisis centers Out-patient	Psychologists, ED physician
Wright et al., 2021 [134]	United States	Pilot/Feasibility trial	-Verify methods for assessing adolescents and young adults who had signs or symptoms of depression or suicide ideation and for training professionals to implement mental health interventions using telehealth devices	Teenage and young adult patients prescribed lifelong home parental nutrition (HPN) infusions	University of Kansas Medical Center Out-patient	A total of 4 professionals. They had either PhD and extensive telehealth experience. The other professionals involved were a master's prepared pediatric nurse observer, and home parental nutrition counsellor, and a mental health nurse specialist. The psychologist and nurse mental health specialist were experienced in managing suicide ideation, and mood disorders, and discussing sensitive topics with adolescents

*TMF* Theory, Model, Framework, *ED* Emergency department

### What ICT-based interventions for suicide prevention have been implemented in clinical settings?

This review identified a total of 66 ICT-based interventions for suicide prevention implemented in clinical settings. Based on the WHO levels of suicide prevention strategies, identified ICT-based interventions were used as universal (*n* = 4), selective (*n* = 10), or indicated (*n* = 53) strategies for suicide prevention. One ICT (i.e., Virtual Hope Box app) was used as both selective and indicated strategies in different studies [69, 88, 109]. While most ICTs targeted individuals who were at an imminent risk of suicide or were displaying early signs of suicide potential, fewer ICTs were used as selective strategies targeting at-risk populations, such as veterans, or patients living with human immunodeficiency virus, or cancer. A few ICT-based interventions were used as universal strategies aimed at population level, which may be explained by this review’s inclusion criteria being clinical context.

The 66 ICT-based interventions for suicide prevention served multiple functions; they were used for suicide screening and assessment (*n* = 22), safety planning (*n* = 20), lethal means restrictions and/or counselling (*n* = 3), discharge or post-discharge follow-up care (*n* = 27), therapy such as dialectical behavior therapy (*n* = 4), and additional resources such as wellness tips and journals (*n* = 18). Other (*n* = 12) functions of ICTs included reminders to appointments, care plans, and peer supports. Following the MHCC typology, most of the ICTs were categorized as computerized interventions (e.g., web-based tools), resources, and applications (*n* = 55), of which 11 were text messages, 10 were mobile applications (apps), and two were emails. Other types included telehealth and telemedicine (*n* = 16), wearable computing and monitoring (*n* = 1), virtual reality (*n* = 2), peer support through social media (*n* = 2), and a robot (*n* = 1) (i.e., chatbot). Table 3 summarizes above characteristics of ICT-based interventions for suicide prevention.

**Table 3:** Characteristics of included ICTs

Target age	ICT intervention	References	Suicide Prevention Intervention Category							MHCC Typology						WHO category		
			Screening and Assessment	Safety Planning	Lethal Means Restrictions and/or Counselling	Discharge or Post-Discharge Follow-Up	Therapy	Resources	Other	Computerized interventions, resources, and applications	Telehealth and telemedicine	Wearable computing and monitoring	Virtual reality	Peer support through social media and other technologies	Robots	Universal	Selective	Indicated
**Total counts (n)**	**66**	**75**	**22**	**20**	**3**	**27**	**4**	**18**	**12**	**55**	**16**	**1**	**2**	**2**	**1**	**4**	**10**	**53**
Adult	Addiction Comprehensive Health Enhancement Support System (ACHESS)	Mackie et al., 2017 [102]		**✓**		**✓**		**✓**		**✓**	**✓**			**✓**				**✓**
16-25yrs	Affinity	Bailey et al., 2020 [67]						**✓**	**✓**	**✓**				**✓**				**✓**
Adult	ALGOrithm for Suicide prevention (ALGOS) Telephone contact	Vaiva et al., 2018 [86]				**✓**					**✓**							**✓**
		Vaiva et al., 2011[65]																
Youth	As Safe as Possible (ASAP)	Kennard et al., 2018[121]		**✓**		**✓**				**✓**	**✓**							**✓**
	BRITE (SMS)			**✓**				**✓**	**✓**	**✓**								**✓**
Youth	Ask Suicide Screening Questions (ASQ) via tablet	Pickett et al., 2021[132]	**✓**							**✓**						**✓**		
Adult	BackUp	Nuij et al., 2018[129]		**✓**						**✓**								**✓**
	mEMA app		**✓**							**✓**								**✓**
Youth	Be Safe App	Gregory et al., 2017 [93]		**✓**						**✓**							**✓**	
Adult	BEACON (Smartphone assisted problem solving therapy)	Hatcher et al., 2020 [74]	**✓**					**✓**		**✓**								**✓**
Youth	BeyondNow app	Muscara et al., 2020 [128]		**✓**				**✓**		**✓**								**✓**
≥ 16yrs		Melvin et al., 2019 [126]																
Youth	BlueIce	Stallard et al., 2018 [84]						**✓**		**✓**								**✓**
		Stellard et al., 2016 [64]																
		Grist et al., 2018 [101]																
		Muscara et al., 2020 [128]																
≤ 19yrs	Brake of My Mind (BoMM)	Jeong et al., 2020 [119]		**✓**				**✓**		**✓**								
≥ 15yrs	Brief Mobile Treatment (BMT) (SMS)	Marasinghe et al., 2012 [79]				**✓**		**✓**		**✓**								**✓**
Adult	Care management intervention	Simon et al., 2016 [63]	**✓**			**✓**				**✓**	**✓**							**✓**
	Skills training intervention								**✓**	**✓**								**✓**
Adult	Caring contacts via text message	Comtois et al., 2019 [71]				**✓**				**✓**	**✓**							**✓**
Adult	Caring letters (email)	Luxton et al., 2012 [124]		**✓**		**✓**				**✓**								**✓**
		Luxton et al., 2014 [62]																
		Luxton et al., 2020 [78]																
Youth	Child Health Improvement through Computer Automation (CHICA) system	Etter et al., 2018 [90]	**✓**						**✓**	**✓**						**✓**		
Adult	Computer interview/self-rating modification of the Hamilton Rating Scale for Depression	Levine et al., 1989 [97]	**✓**							**✓**								**✓**
Adult	Computerized Self-Reported patient-reported outcome (PRO) Assessment	Lawrence et al., 2010 [96]	**✓**							**✓**							**✓**	
Youth and their parents	Crisis Care (Web-based)	McManama O'Brien et al., 2017 [125]				**✓**		**✓**		**✓**								**✓**
≥ 16yrs	e-DASH (electronic—Depression and Self-Harm)	Sayal et al., 2019 [133]					**✓**			**✓**	**✓**							**✓**
Youth	Emotion regulation individual therapy for adolescents (ERITA)	Morthorst et al., 2021 [127]					**✓**		**✓**	**✓**								**✓**
Youth	Empatica E4 (Empatica Srl)	Kleiman et al., 2019 [122]	**✓**									**✓**						**✓**
Adult	Enhanced electronic suicidality alert system	Madan et al., 2015 [98]	**✓**							**✓**							**✓**	
Youth	Internet-based Emotion Regulation Individual Therapy for Adolescent (ERITA)	Olsen et al., 2021[130]				**✓**				**✓**								**✓**
Adult	Jaspr Health	Dimeff et al., 2021 [115]	**✓**	**✓**	**✓**			**✓**	**✓**	**✓**					**✓**			**✓**
Adult	LifeApp’tite Mobile App	O'Toole et al., 2019 [82]		**✓**		**✓**		**✓**	**✓**	**✓**								**✓**
Adult	Lock to Live (L2L)	Betz et al., 2020 [106]			**✓**					**✓**								**✓**
Youth	MEDIACONNEX (SMS)	Ligier et al., 2016 [77]				**✓**				**✓**								**✓**
Youth	MI-SafeCope	Czyz et al., 2021[113]				**✓**					**✓**							**✓**
	Post discharge text-based support (Texts)					**✓**				**✓**								**✓**
Adult	Mobile Messenger Counselling Services (MMC)	Seong et al., 2021 [99]				**✓**					**✓**							**✓**
Adult	Mobile telephone message interventions	Chen et al., 2010 [111]				**✓**				**✓**								**✓**
Adult and Youth	MyPlan	Andreasson et al., 2017 [66]		**✓**				**✓**		**✓**								**✓**
		Buus et al., 2020 [100]															**✓**	
10-24yrs	New Hope	O'Keefe et al., 2019 [81]		**✓**						**✓**								**✓**
	Optimized Care Management		**✓**							**✓**								**✓**
Adult	Online assessment of suicidality in patients with cancer	Kolva et al., 2020 [94]	**✓**							**✓**							**✓**	
Adult	Online dialectical behavior therapy skills	Simon 2022 [83]	**✓**			**✓**	**✓**			**✓**								**✓**
14-25yrs	Online tool for self-monitoring of depression and suicidal ideation	Hetrick et al., 2017[117]	**✓**							**✓**							**✓**	
Adult	Post-acute crisis text messaging outreach, Suicide intervention assisted by messages (SIAM)	Berrouiguet and Gravey et al., 2014 [105]				**✓**			**✓**	**✓**								**✓**
		Berrouiguet and Alavi et al., 2014 [61]																
		HUGOPSYNetwork et al., 2018 [68]																
Adult	Project Life Force Safety Planning Mobile Apps	Goodman et al., 2020 [73]		**✓**						**✓**								**✓**
Adult	SAFETEL	O’Connor 2019 [135]		**✓**		**✓**				**✓**	**✓**							**✓**
Adult	SafeTy and Recovery Therapy (START) -Follow up Telephone Coaching and Mobile augmentation	Depp et al., 2021 [114]				**✓**		**✓**	**✓**	**✓**	**✓**		**✓**					**✓**
≥ 16yrs	SMS Text Messaging- SMS SOS Study	Stevens et al., 2019 [85]				**✓**				**✓**								**✓**
Not reported	Stop Depression platform	Cassola et al., 2017 [110]	**✓**						**✓**	**✓**						**✓**		
Adult	Strength Within Me (SWiM)	Bruen et al., 2020 [108]		**✓**				**✓**		**✓**								**✓**
Adult	Suicide intervention via home-based telehealth	Gros et al., 2011[103]		**✓**	**✓**						**✓**						**✓**	
Adult	Suicide interventions sent via mobile phone text messaging technologies	Kodama et al., 2016 [123]				**✓**				**✓**								**✓**
Youth	Suicide risk detected via adolescent depression screening	Davis et al., 2021 [89]	**✓**							**✓**								**✓**
Youth	TeenTEXT	Owens et al., 2016 [131]		**✓**						**✓**								**✓**
Youth and young adults	Telehealth distance health care	Wright et al., 2021 [134]	**✓**					**✓**		**✓**							**✓**	
Adult	Telehealth monitoring system using Health Buddy	Kasckow et al., 2015 [76]	**✓**			**✓**					**✓**							**✓**
Adult		Kasckow et al.,2016 [120]																
Adult	Telephone contact	Vaiva et al., 2006 [87]				**✓**					**✓**							**✓**
≥ 15yrs	Telephone follow up	Mousavi et al., 2014 [80]				**✓**					**✓**							**✓**
All ages	Telephone management programme	Cebrià 2013[70]				**✓**					**✓**							**✓**
Youth	TextIt	Czyz et al., 2020 [112]		**✓**		**✓**		**✓**		**✓**								**✓**
Adult	The Parkland Health & Hospital System (PHHS) Universal Suicide Screening Program	Canady 2018 [104]	**✓**							**✓**						**✓**		
Youth	The Safety Planning Assistant	Hill et al., 2020 [118]		**✓**						**✓**							**✓**	
Adult	The tailored Men and Providers Preventing Suicide (MAPS) program	Jerant et al., 2020 [75]	**✓**							**✓**								**✓**
Adult	True Colours online questionnaire (digital self-monitoring component)	Brand et al., 2021[107]	**✓**							**✓**								**✓**
Adult	Virtual Collaborative Assessment and Management of Suicidality System (V-CAMS)	Dimeff et al., 2020 [116]	**✓**					**✓**	**✓**	**✓**			**✓**					**✓**
Adult	VigilanS (renamed after ALGOS)	Duhem et al., 2018 [72]				**✓**					**✓**							**✓**
		Fossi Djembi et al., 2020 [91]																
		Fossi et al., 2021[92]																
Adult	Virtual Hope Box (VHB)	Bush et al., 2015 [109]		**✓**				**✓**		**✓**							**✓**	
		Bush et al., 2017 [69]																**✓**
		Chen et al., 2018 [88]																
Adult	Virtual Monitoring	Kroll et al., 2020 [95]							**✓**	**✓**								**✓**

Adult: Above 18, Youth: Below 18

### Implementation strategies

Overall, there was a lack of reporting on the implementation strategies for the included ICTs. Of the 75 included papers, 31 reported implementation strategies, but the level of detail varied. Training clinicians (*n* = 15) was the most commonly reported implementation strategy for the new ICT, focusing on building new skills [62, 63, 70, 71, 75, 95, 101, 110, 114, 115, 121, 127–129, 134]. A few studies specifically reported using demonstration [101] and simulation methods for training [115]. Educational meetings or communication (e.g., phone, email) (*n* = 12) was the next common implementation strategy which provided clinicians with new information and/or instructions required for the ICTs [73, 74, 84, 93, 101, 104, 108, 114, 126, 128, 131, 135]. Education or training were sometimes accompanied by educational materials (e.g., written handouts or supportive tools like a pocket guide) (*n* = 6) [64, 73, 101, 110, 114, 131]. Training and education were made distinct in this review; training focused on building practical skills, whereas education focused on providing new information or knowledge. Eight studies reported collaborative initiatives with clinicians, Information Technology (IT) consultants, ministry, institutions and/or managers [74, 91, 94, 95, 114, 123, 124, 128]. For example, collaboratives initiatives involved nominating site staff as co-principal investigators [74], or consulting key stakeholders before the start of the study [123]. Six reported providing ongoing supervision for using the ICT [63, 67, 71, 72, 104, 127], of which one study specifically conducted audits and provided daily reports to unit managers and nursing leaders [104]. Three studies provided opportunities for clinicians to participate in discussion for improvement in the implementation of the ICT, contributing to iterative changes in the implementation process during the study [67, 95, 114]. Two studies reported tailored approaches to implementation; one created a new clinical workflow to ensure that the implementation was seamless and minimized interruptions by leveraging existing staff roles and processes as much as possible [132], and the other provided site-specific training [62]. Lastly, one study provided onsite technical IT support [104].

### What are the reported barriers and facilitators to implementing these ICT-based interventions?

Overall, there was a general lack of reporting on barriers and facilitators to implementation. Nineteen studies reported several barriers and/or facilitators with a varying level of detail. Barriers and facilitators that were most frequently reported by identified studies were associated with *physical* (*n* = 12) or *social* (*n* = 10) *opportunity* within the COM-B/TDF. TDF domains for physical (i.e., external) opportunities include environmental context and resources, whereas social opportunities include the social influences, such as norms and cultural factors [51]. Internet instability [134], limited telephone lines [103], lack of patients’ access to smart devices [107], time limited nature of clinical settings [76, 82, 102, 131], and no access to research teams to troubleshoot technological issues [108] were physical barriers described in the included studies. Other physical barriers included administrative challenges such as hospital policy that did not allow patients to use smartphones in the in-patient settings [93]. Therefore, even if patients had their own devices, hospital policy or the discharge norms limited patients’ access and did not allow enough opportunity for clinicians to deliver the ICTs until the moment of discharge. This not only speaks to physical barriers (i.e., hospital policy), but also reflects social barriers of limiting ICT related interactions with patients [93]. Other barriers to implementing ICTs related to social opportunity included lack of engagement with clinicians in the study and lack of buy-in and support from the clinicians [128, 131, 132]. Some of the facilitators were the direct opposite of barriers. In contrast to lack of engagement with clinicians, positive working collaborations between clinicians and the research team facilitated the implementation process [62, 104, 108, 131]. For example, one study had a hospital staff member in the role of principal investigator at each study site [62]. Furthermore, leadership engagement, such as manager approvals for implementation, facilitated ICT implementation, and some managers insisted on circulating implementation information to clinicians via e-mail [131].

*Reflective* (*n* = 14) and *automatic* (*n* = 3) *motivations* were the next commonly coded barriers and facilitators in this review. Motivation encompasses all brain processes that direct behaviour [49]. This includes not just reflective motivation, such as goals, analytical and conscious decision-making that leads to behaviour, but it also includes autonomic motivation like habits and emotional responses [49]. Reflective motivation includes TDF domains of professional roles and identities, beliefs about consequences, beliefs about capabilities, optimism, intentions and goals [51]. Defining roles and responsibility attributes [108], perceived burdens, and uncertainties associated with ICTs [76, 82, 131] were examples of barriers noted among the reflective motivation category. For example, clinicians were worried about ICT devices being stolen or broken [108] and perceived that that the ICT may have a better fit in other, non-clinical settings such as schools [131]. Clinicians also did not appreciate the perceived burdens of implementing ICTs because introducing new ICTs possibly created new tasks, taking extra time in their usual clinical flow [76, 82]. When clinical settings included multi-disciplinary teams, clinicians were concerned about who should be responsible for the ICT, but identifying appropriate professional roles and having designated staff for the new ICT were reported facilitators [78, 104, 108]. For example, one study implemented caring emails as post-discharge follow-up care for suicide prevention and reported that the new task associated with this ICT could be reasonably done by existing hospital staff rather than hiring new staff [78]. Additionally, they reported minimal requirements for clinicians to manage the new ICT, which facilitated implementation [78]. In contrast to uncertainties around ICTs, perceived benefits and usefulness of ICTs were facilitators [102, 109]. Automatic motivation refers to the TDF domain of emotion [51]. Negative (“technophobia”) or positive outlook about the ICTs [102, 116, 131] were identified as barriers or facilitators.

Implementation barriers and facilitators related to *psychological* (*n* = 14) *capabilities* were the least frequently coded category. Psychological capabilities include one’s knowledge, memory, and ability to make decisions and regulate behaviours [54]. Identified papers reported barriers and facilitators related to the knowledge and skills about ICTs, awareness of necessary resources, and clinicians’ cognitive load. For example, having no manual or guidelines to instruct clinicians on how ICTs should be introduced to patients and used for suicide prevention treatment was a barrier [82, 107]. In contrast, training resources and education sessions were facilitators that helped to build clinicians’ psychological capabilities [104, 109, 116, 134]. Additionally, a few ICTs helped to decrease clinicians’ cognitive burden [116, 131]. A summary of the COM-B/TDF analysis can be found in Table 4, and a full breakdown of extracted and analysed data can be found in Additional file 4.

**Table 4:** Barriers and facilitators to implementing ICTs

COM-B	TDF Domains	Definitions	Frequency of occurrence	Examples of barriers and facilitators
Capability	Knowledge	An awareness of the existence of something	7	• Educating staff about the reasons for universal screening prior to implementation (Facilitator) • No manual or guidelines as to how the mobile app should be introduced and used throughout treatment (Barrier)
	Skills	An ability or proficiency acquired through practice	5	• No access to appropriate training to ensure that nurses feel able to use innovative technology (Barrier) • Training health care professionals for assessing and caring for patients from a distance using mobile telehealth iPad interactions (Facilitator)
	Memory, attention and decision processes	The ability to retain information, focus selectively on aspects of the environment and choose between two or more alternatives	2	• Clinical decision support tool to provide a definitive recommendation about whether to hospitalize or release a patient, decreasing cognitive burden (Facilitators)
Motivation	Social/professional role and identity	A coherent set of behaviours and displayed personal qualities of an individual in a social or work setting	6	• Concerned for who [which clinician] would be responsible for monitoring the devices (Barrier) • Task can be reasonably done by existing hospital staff and the minimal requirement to manage replies from participants who were in crisis (Facilitator)
	Optimism	The confidence that things will happen for the best or that desired goals will be attained	2	• Clinicians and managers agreed that the new ICT made sense and was immediately appealing (Facilitator)
	Beliefs about Consequences	Acceptance of the truth, reality, or validity about outcomes of a behaviour in a given situation	5	• Uncertainty about how well the mobile app was incorporated in the face-to-face treatment, and whether this led to a positive or negative effect (Barrier) • Concerns about giving service users iPhones and Fitbits for the duration of the study, suggesting that the equipment would either be stolen or damaged (Barrier) • Perceived value or benefit of the tool to help patients (Facilitator)
	Goals	Mental representations of outcomes or end states that an individual wants to achieve	1	• Perception that the intervention may have better fit with schools and universal youth services (Barrier)
	Emotion	A complex reaction pattern, involving experiential, behavioural, and physiological elements, by which the individual attempts to deal with a personally significant matter or event	3	• Perceived burdensomeness and technophobia (Barrier)
Opportunity	Environmental context and resources (Physical)	Any circumstance of a person’s situation or environment that discourages or encourages the development of skills and abilities, independence, social competence and adaptive behaviour	12	• Occasional dropped or slow connections, pixel blurring, and the need for online security (Barrier) • Taking extra time away from the usual therapy (Barrier) • Using the smartphone application was more time consuming (Barrier) • No access to the research team available in participating wards to troubleshoot technological issues in a timely manner (Barrier) • Patients' lack of access to the technology (e.g., smart phones) (Barrier) • Inexpensive ICT (Facilitator)
	Social influences	Those interpersonal processes that can cause individuals to change their thoughts, feelings, or behaviours	10	• Having a hospital staff member in the role of principal investigator at each site (Facilitator) • Positive working collaborations between clinicians and the research team, including data scientists and technicians, to ensure a continuous flow of data (Facilitator) • Waiting to download the app until the moment of discharge limits the opportunity for staff to facilitate the adoption of a smartphone app (Barrier) • Limited buy-in at management levels (Barrier)

### What are the reported measures and outcomes of these ICT-based interventions?

**Fig. 2:**
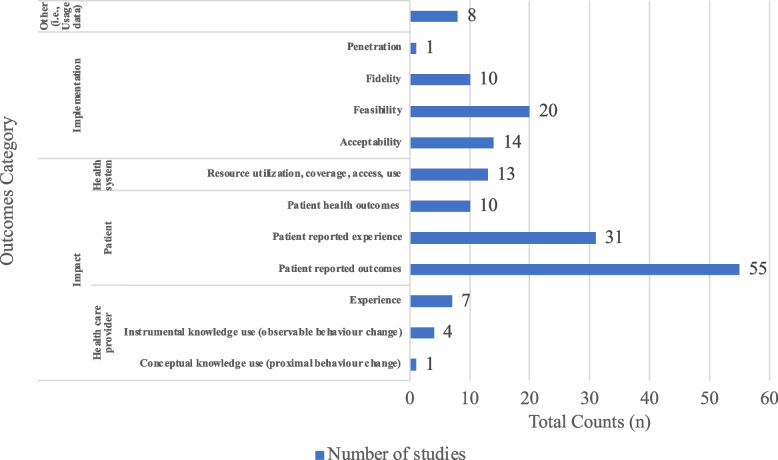
Reported outcome types

As shown in Fig. 2, studies reported PRO (*n* = 55), PRE outcomes (*n* = 31), and patient health outcomes (e.g., mortality) (*n* = 10). Examples of PRO included assessing patients’ suicide ideation, suicide risk, coping ability, depressive symptoms, and health-related quality of life using validated tools such as Beck Scale for Suicide Ideation, Patient Health Questionnaires, Columbia Suicide Severity Rating Scale, and Beck Depression Inventory. Examples of PRE outcomes included assessing overall experiences and perceptions of ICTs, patient satisfaction, engagement with ICTs using open-ended survey questions, Likert-scale surveys, written feedback, or interviews. Patient health outcomes such as mortality and adverse events often came from health administrative data, electronic health records, or insurance claim data. At health care provider-level outcomes, studies reported clinician experiences (*n* = 7), clinicians’ instrumental knowledge use (*n* = 4), such as number of documented referrals, and conceptual knowledge use (*n* = 1), such as professional knowledge about suicide. Thirteen studies reported health system-level outcomes such as readmission rates and medical costs. Additionally, eight studies specified usage data as an outcome of interest.

Following Proctor’s definitions for implementation outcomes [56], studies reported feasibility (*n* = 20), acceptability (*n* = 14), fidelity (*n* = 10), and penetration (*n* = 1) of the ICTs. Feasibility outcomes included perceived compatibility of ICTs in the clinical settings or practicality of ICTs assessed by surveys, open-ended questionnaires, interviews or measuring the time required to complete the ICT-related module. Acceptability of ICTs was evaluated by user experience, perception, agreeableness, or satisfaction using surveys, open-ended questionnaires, or interviews. Fidelity outcomes included the completion of follow-ups and/or adherence to treatments using chart reviews or self-reported data. Penetration was measured by the proportion of people who attempted suicide and were enrolled in an ICT-based intervention (i.e., VigilanS) relative to all included samples of people who attempted suicide regardless of their enrollment. None of the studies reported adoption, appropriateness, implementation cost, or sustainability outcomes of implementation. See Table 5 for summaries of the outcomes of interest, outcome measures, measurement tools, and key results of the 70 included studies.

**Table 5:** Summary of the outcomes, measures, and key results

Author, Year	Outcomes of interest	Outcome measures and measurement tools	Key results
Andreasson et al., 2017 [66]	Suicide ideation, hopelessness, depressive symptoms, and app/user satisfaction	• Beck Suicide Ideation Scale • Beck hopelessness scale • Major depression inventory • Client satisfaction questionnaire	Not Applicable—protocol
Bailey et al., 2020 [67]	Suicidal ideation, depression, perceived burdensomeness and thwarted belongingness, social connectedness, mindfulness, self-compassion, problem-solving, suicide attempts, self-harm, feasibility, and acceptability	• Adult Suicidal Ideation Questionnaire • Patient Health Questionnaire—9 • 15-item version of the interpersonal needs questionnaire • Social connectedness scale—revised • Mindful attention awareness scale • Self-compassion scale – short form • Negative problem orientation questionnaire • Columbia Suicide Severity Rating Scale • Deliberate self-harm inventory • Usage data and activity data	Overall, more than half of the participants logged in at least once per week satisfying this criterion related to acceptability. There was also significant variability in Café activity (including posts, replies, and likes/reactions), steps and actions completed, and amount of user-initiated contact with moderators
Berrouiguet and Alavi et al., 2014 [61] (protocol) HUGOPSY Network et al., 2018 [68]	Suicide reattempt, suicide deaths, suicide ideation, medical costs, and satisfaction	• Columbia Suicide Severity Rating Scale • Medico-economic questionnaire Satisfaction questionnaire • Mini-international neuropsychiatric interview • Narrative description of circumstances associated with their participant-initiated contact	In each case, the contact has been initiated by the study participant immediately after receiving a message (Case 3) or a few days later (Case 1 and 2). These cases highlight the potential for connecting individuals to crisis services after an SA using automated text messages. This text message-based brief contact intervention has demonstrated the potential to reconnect suicidal individuals with crisis support services while they are experiencing suicidal ideation as well as in a period after receiving messages
Berrouiguet and Gravey et al., 2014 [105]	Feasibility, acceptability	• Text messages status reports and the transmission rates issued by the web server engine • Standardized phone interview and questionnaire	Receiving text messages sent from an intranet program after a suicide attempt is technically possible. This post-crisis outreach program was accepted by the patients who found it to have a positive preventive impact
Betz et al., 2020 [106]	Feasibility, acceptability, suicide severity	• Minutes for the patient to complete L2L and the completion rate • Ottawa acceptability scale • Decisional conflict scale • Columbia Suicide Severity Rating Scale	The L2L decision aid appears feasible and acceptable for use among adults with suicide risk and may be a useful adjunct to lethal means counseling and other suicide prevention interventions
Brand and Hawton 2021 [107]	Patients' and nurses' experiences	• Questionnaire (Likert-scale questions, binary questions, and open-ended questions)	All the participants who attended more than two sessions (*n* = 8) found the weekly True Colours questionnaires easy to use. Four of the five nurses who participated in the evaluation stated that they found it easy to recruit patients and explain the benefits of True Colours to them. The remaining nurse found registering a patient onto the True Colours system challenging. All the nurses who used True Colours found it useful
Bruen et al., 2020 [108]	Acceptability, engagement	• Fitbit data • Brief informal exit interview	A total of 61 safety plans were completed, with an average of 2.5 plans per person. SWiM App was helpful: The ability to write-out thoughts suited those people who might otherwise have had to struggle to voice these verbally. Most participants provided positive responses about using Fitbit, which included increased self-awareness of levels of physical activity, goal setting, and peer motivation
Bush et al., 2015 [109]	Patients' and clinicians' experiences	• Semi structured interview • Self-report questionnaires (e.g., Likert-type rating scale) • Electronic usage log	High-risk patients and their clinicians used the VHB more regularly and found the VHB beneficial, useful, easy to set up, and said they were likely to use the VHB in the future and recommend the VHB to peers
Bush et al., 2017 [69]	Coping, suicide ideation, reasons for living	• Coping self-efficacy scale • Beck Scale for Suicidal Ideation • Brief reasons for living inventory	VHB users reported significantly greater ability to cope with unpleasant emotions and thoughts at three and 12 weeks compared with the control group. No significant advantage was found on other outcome measures for treatment augmented by the VHB
Buus et al., 2020 [100]	Patients' experience	• Focus group	Users found that the MYPLAN app was helpful for learning to recognize early signs of an impending crisis, and for coping by actively finding personalized problem-solving strategies. This study indicates that there were huge variations in users’ engagement and use of MYPLAN
Cassola et al., 2017 [110] (Protocol with preliminary results)	Satisfaction and usability	• System usability scale questionnaire	System usability surveys reveal that users were pleased with the use of the system during the Stop Depression clinical trials. Qualified users considered the platform to be straightforward and with a low learning curve, having felt confident while using it. Moreover, an extremely high percentage of users claimed that they would use the system frequently
Cebrià et al., 2013 [70]	Suicide attempt and reattempt	• Telephone survey	The results obtained suggest that the application of a telephone management programme to patients discharged from an emergency room for suicide attempts significantly delays further attempts and decreases the rates of reattempts in the context of a general reduction
Chen et al., 2010 [111]	Patient experience, suicide attempt	• Interview	Mobile telephone text message interventions could be a feasible and acceptable follow-up method with suicide attempters. All suicide attempters in our sample who were seen in emergency departments have access to a mobile telephone, and there is no charge for the user to receive text messages. After four message contacts, most of them thought it was acceptable and said they would like to receive the messages for a longer time
Chen et al., 2018 [88]	Suicide ideation, coping, app usage	• App usage logs • Beck Scale for Suicidal Ideation • Coping self-efficacy scale	Older age was correlated with higher levels of usage. Participants who had 2 years or more of college had lower levels of VHB usage. The findings suggested a potential association between usage and efficacy for stopping negative thoughts. Usage was associated with increased efficacy for stopping negative thoughts, though this relationship was attenuated among participants with high levels of usage
Comtois et al., 2019 [71]	Suicide ideation, suicide attempt, ED visit	• Suicide status interview • Treatment history interview • Suicide attempt self-injury count • Hurdle model	There was no significant effect on the likelihood or severity of current suicidal ideation or likelihood of a suicide risk incident; there was also no effect on emergency department visits. However, participants who received Caring Contacts had lower odds than those receiving standard care alone of experiencing any suicidal ideation between baseline and follow-up and fewer had attempted suicide since baseline in the group receiving Caring Contacts vs the standard-care group
Czyz et al., 2020 [112]	Hopelessness, positive and negative affect, patient perceptions and experiences with messages	• Daily survey • 10-item positive and negative affect • Schedule for children • Open ended feedback	Quantitative and qualitative feedback across the 2 study phases pointed to the acceptability of text-based support
Czyz et al., 2021 [113]	Suicide ideation, self-efficacy, coping, suicide attempt, suicide injury, safety plan use	• Efficacy to cope with suicidal parental self-efficacy scale thoughts and urges scale • Columbia Suicide Severity Rating Scale • Self-assessed expectations of suicide risk scale • Non-suicidal self-injury portion of the self-injurious thoughts and behaviors interview	The results from this pilot study suggest that study procedures for optimizing interventions for adolescents at elevated suicide risk were feasible and acceptable. Moreover, results indicate that specific intervention components and sequences influenced key mechanisms of change and have potential to reduce risk of suicidal behavior
Davis et al., 2021[89]	Suicide risk, fidelity of screening process	• Patient Health Questionnaire (PHQ) – modified for teens • Columbia diagnostic interview • Schedule for children-depression scale • Manual chart review	The study results indicated the high degree of fidelity to the follow-up suicide risk questions. Follow-up: suicide-specific follow-up actions were relatively sparse in the year following PHQ-9-M screening per a retrospective manual chart review
Depp et al., 2021[114]	Suicide ideation, suicide behaviour, satisfaction, service utilization, acceptability, adherence, and fidelity	• Scale for suicide ideation or Columbia Suicide Severity Rating Scale • Outpatient follow-up interval • Composite suicide-related crises • Ecological Momentary Assessment adherence or outcomes • Tablet routines questionnaire • Brief psychiatric rating scale • Treatment rationale scale • Timeline follow back scale • Intervention satisfaction questionnaire	Not Applicable—protocol
Dimeff et al., 2020 [116]	Feasibility	• Semi structured interview • Usability satisfaction and acceptability questionnaire ratings • Open ended qualitative data from Dr. Dave (Artificial Intelligence avatar)	Technology tools including a patient-facing avatar and e-caring contacts, along with provider-facing tools may offer a powerful method of facilitating best-practice suicide prevention interventions and point-of-care tools for suicidal patients seeking ED services and their medical provider
Dimeff et al., 2021 [115]	Coping, patient experience, adverse events, acceptability, feasibility	• Safety and imminent distress questionnaire • Suicide-related coping scale • Jaspr health patient satisfaction questionnaire • Brief semi structured interview	Of 14 Jaspr Health patients, all completed a comprehensive suicide assessment and created a crisis stabilization plan, and 12 (85%) patients engaged in lethal means counseling. Jaspr Health participants also opted to learn 3 behavioral skills and gave Jaspr Health high satisfaction ratings. In addition, no adverse events occurred during its use. Jaspr Health appeared clinically effective
Duhem et al., 2018 [72]	Professional knowledge about suicide, suicide attempt, health care pathway, acceptability, fidelity	• Regional suicide mortality data • Penetrance rate • Quantitative appraisal (digital survey) • Qualitative appraisal (semi structured interviews) • Two-step medico economic assessment of the programme • Crisis card measures	Not Applicable—protocol
Etter et al., 2018 [90]	Provider follow-up action, suicide risk, depression, substance use	• A single question based on American Academy of Pediatrics • Patient Health Questionnaire—2 • Chart abstraction (provider worksheet)	Incorporating adolescent suicide screening and provider follow-up guidance into an existing computer decision support system in primary care is feasible and well utilized by providers
Fossi Djembi et al., 2020 [91]	Suicide attempt, penetration of VigilianS	• VigilanS database • Health administrative data	Twenty-one centers were running VigilanS in 2018, with an average penetrance of 32%. A significant relationship was identified, showing a sharp decrease in suicide attempt as a function of penetrance
Fossi et al., 2021 [92]	Suicide reattempt	• Second entry in VigilanS	Findings suggests the effectiveness of VigilanS on suicide reattempt, from the first entry into VigilanS. Maintaining contact is of great importance for the patient’s future
Goodman et al., 2020 [73]	Suicide behaviour, depression, hopelessness, coping and treatment utilization	• Medical record abstraction • Brief safety plan scoring form • Columbia Suicide Severity Rating Scale	Not Applicable—protocol
Gregory et al., 2017 [93]	Smartphone ownership, app usage, admission to hospital	• Questionnaires/surveys	Of the 76 patients, 50 reported that they owned a smart phone. Of the 26 who reported they did not own a smartphone, five patients reported that they still intended to download the Be Safe app later. Of the 50 patients who owned a smartphone, nine downloaded the Be Safe app in hospital. Of the 41 smartphone owners who did not download in hospital, 34 stated they intended to download the app later, and four additional patients stated they would “maybe” download the app later. Fifty-one out of 74 patients were on their first admission to hospital
Grist et al., 2018 [101]	Usability, acceptability, safety	• Interview	6 key themes emerged: (1) appraisal of BlueIce,(2) usability of BlueIce, (3) safety (4) benefits of BlueIce, (5)agency and control, and (6) BlueIce less helpful. Overall, BlueIce was deemed to be helpful, easy to use, and safe
Gros et al., 2011 [103]	Efficacy and symptoms	• Beck's depression inventory -2 • Beck anxiety inventory • Post-traumatic stress disorder (PTSD) checklist – military version	The preliminary findings in the present case support the use of telehealth in the identification and intervention of suicidality at home
Hatcher et al., 2020 [74]	Suicide ideation, depression, anxiety, PTSD symptoms, meaning in life, social support, quality of life, substance use, health service use, app usage	• Beck Scale for Suicide Ideation • Patient Health Questionnaire • Generalized anxiety disorder 7-item scale • Post-traumatic stress disorder (PTSD) screen • EuroQol 5-Dimension 5-level questionnaire • Experienced meaning in life scale • Multidimensional scale of perceived social support • Alcohol use disorders identification test • Drug abuse screening test • Administrative health data • Smartphone application usage data • Interviews	Not Applicable—protocol
Hetrick et al., 2017 [117]	Feasibility, acceptability, perceived usefulness, depression	• Questionnaire about acceptability and usefulness including open-end-ed questions • Suicidal Ideation Questionnaire – junior • Patient Health Questionaries—9	The e tool was feasible to implement. Young people and clinicians found the tool acceptable and useful for understanding symptoms and risk
Hill et al., 2020 [118]	Depression, suicide ideation, satisfaction, patient experience, acceptability, feasibility	• Time required to complete the module • Safety plan completion • Feedback form (open-ended questions) satisfaction • Short mood and feelings questionnaire • Suicide ideation questionnaire- junior	Adolescents’ reported satisfaction with the intervention was high at both post-treatment and follow-up. At the follow-up assessment, 11 of the 15 adolescents reported using their safety plan, of whom 8 (72.7%) found their safety plan to be helpful and 7 (63.6%) reported that their safety plan prevented them from making a suicide attempt. The average time to complete the adolescent safety plan module was 48.13 min. Data support the preliminary feasibility of administering safety planning using the web-based tool and the acceptability of the Safety Planning Assistant
Jeong et al., 2020 [119]	Attitudes, behaviour control, suicide attempts, user experience	• System usability scale questionnaire	Study 1: Results indicated no usability problems or minimal usability problems with a low priority for revision, and the level of usability of BoMM is acceptable. Study 2: In all participants, attitude toward suicide attempts declined at each of the three time points
Jerant et al., 2020 [75]	Whether the topic of suicide was discussed during the visit, suicidal thought, suicide risk	• Beck Scale for Suicide Ideation • Patient Health Questionnaire • Primary care PTSD screen • Alcohol use disorder identification test	Any suicide discussion was more likely among the tailored Men and Providers Preventing Suicide (MAPS) patients than controls. In the examination of moderation of the intervention effect by the presence or absence of any suicide preparatory behaviors, the interaction effect was not statistically significant
Kasckow et al., 2015 [76]	Suicide ideation, depression, feasibility	• Suicide severity interview • Beck Scale for Suicide Ideation • Calgary depression rating scale • Percentage of days active participants downloaded responses to the questions	Daily adherence in the use of the Health Buddy (HB) system during months 1–3 was, respectively, 86.9%, 86.3%, and 84.1%. There were significant improvements in Beck Scale for Suicide Ideation scores in HB participants. There were no changes in depressive symptoms. Telehealth monitoring for this population of patients appears to be feasible
Kasckow et al.,2016 [120]	Suicide ideation, depression, user experience, feasibility, adherence	• Beck Scale for Suicidal Ideation (BSSI) • Calgary depression rating scale • Number of participants accessed the system • Open-ended surveys	Our pilot findings suggest that the use of our telehealth monitoring system is feasible in monitoring post-discharge suicide risk in this population. Monthly adherence for telehealth participants was > 80%. A qualitative analysis of endpoint surveys revealed that most participants had positive responses. In both groups, there were improvements in BSSI scores at endpoint relative to baseline
Kennard et al., 2018 [121]	Suicide ideation, suicide behaviour, treatment utilization, satisfaction	• Columbia Suicide Severity Rating Scale • Suicidal Ideation Questionnaire–junior • Child and adolescent services assessment • Post-study satisfaction and usability questionnaire • Client satisfaction questionnaire-8	Results show acceptability and feasibility of the As Safe as Possible (ASAP) intervention and supporting BRITE app. The RCT was not large enough to detect even substantial clinical effects, but the rates of suicide attempt in those assigned to ASAP/BRITE were half of those in TAU, indicating that this intervention is promising and may have utility in the reduction of post-discharge suicide attempts in hospitalized, suicidal adolescents
Kleiman et al., 2019 [122]	Feasibility, acceptability, user experience	• Survey • Wearable computer comfort rating scale • Usage data • 4 open-ended qualitative question	Results supported the feasibility and acceptability of this approach. Participants wore the monitor for an average of 18 h a day and reported that despite sometimes finding the monitor uncomfortable, they did not mind wearing it
Kodama et al., 2016 [123]	Suicide ideation, social/personnel resources	• Questionnaires (multiple choice questions and Likert scale) outcome data were obtained from participants’ physicians	At the 3- and 6-month time points of the intervention, more than 85% of participants reported that the text messages were helpful or a little helpful. Participants who had committed self-harm during the previous 6 months at baseline accounted for 27.6% of the sample (*n* = 8), whereas the proportion at 6 months significantly decreased. Further, the intensity of suicidal ideation was significantly reduced after the intervention period
Kolva et al., 2020 [94]	Suicide ideation, suicide attempt	• Patient Health Questionnaire (PHQ) • Suicidal behaviors questionnaire—revised (SBQ-R)	Online assessment of suicidality in this sample of adults with heterogeneous cancer diagnoses receiving outpatient psycho‐oncology care was feasible and ethically sound. Active suicidal ideation as identified by the PHQ‐9 was rare, almost all participants denied thoughts that they would be better off dead or active thoughts of self‐harm. Few participants reported having these thoughts for several days or more than half of the days. In contrast, on the SBQ‐R, 28 participants reported thoughts of killing oneself ranging from rarely to very often (*n* = 1, 1.1%) in the previous year
Kroll et al., 2020 [95]	Adverse events and nurse preference for observation	• Software running the monitoring technician's interface with the mobile units automatically logged the information • Free text entered by monitoring technician (MT) • Nursing preference survey	Average daily census for the MTs during the pilot phase was 6.2 patients. The maximum number of patients receiving virtual monitoring for an indication of suicide precautions at a single time was 3. There were no adverse behavioural events. Nurses who did and did not care for patients on virtual monitoring both gave moderately high favourability ratings, and no significant differences in favourability of virtual monitoring or 1:1 between nurses who did and did not care for patients
Lawrence et al., 2010 [96]	Suicide ideation	• Patient Health Questionnaire—9 • Alcohol Use Disorders Identification Test-Concise • Alcohol, Smoking and Substance Involvement Screening Test (ASSIST)	The odds of reporting suicidality were increased with more severe depression and current substance abuse, while advancing age was associated with lower risk. Our experience supports the use of novel technologies and user-friendly interfaces (i.e., touchscreens or tablet computers) to facilitate the collection of self-reported information in high volume clinical settings
Levine et al., 1989 [97]	Self-harm, suicide ideation	• Hamilton rating scale for depression • Suicidal Ideation Questionnaire	Study result suggests that not only is the computer interview acceptable to most patients, but the data suggest that the patients are prepared to confide information to the computer that they may be unwilling to tell the clinician. Further, the data also suggest a significant pathoplastic effect of the personality of the patient on the perception of the psychopathology by the clinician. The computer appeared to be a better predictor of suicidality than the interview by the clinician
Ligier et al., 2016 [77]	Suicide attempt and suicide reattempt	• Data from participating hospital • Multidimensional scale of perceived social support • Kidscreen-27 and Vécu et Santé Perçue des Adolescents • Center for Epidemiologic Studies Depression Scale	Not Applicable—protocol
Luxton et al., 2012 [124]	Feasibility, readmission, length of stay, staff experience, patient coping, depression, suicide ideation, and adverse events	• Reasons for living inventory • Patient Health Questionnaire—9 • Suicide ideation scale • Phone interviews	Most participants indicated preference for e-mail versus postal mail. Fifteen participants were readmitted for treatment compared to 20 patients in usual care. Twenty participants sent responses, and all were positive statements about the program. There were no adverse events. This program is feasible for use at a military treatment facility
Luxton et al., 2014 [62] (Protocol) Luxton et al., 2020 [78]	Suicide mortality, depression, suicide ideation, coping, belongingness, perceived burdensomeness, capability for lethal self-injury, positive aspects in a person's life, suicide behaviour, medical/psychiatric treatment utilization	• Positive assets search semi-structured interview tool • Acquired Capability for Suicide Scale • Patient Health Questionnaire -9 • Lifetime Parasuicide Count • Interpersonal Needs Questionnaire • Acquired Capability for Suicide Scale • Death certificates recorded in the Centers for Disease Control and prevention • National Death Index Plus • Rudd suicide ideation scale • Survey (phone interview) • Health administrative data	No firm conclusions about the efficacy of the intervention can be made because the study was inadequately powered. There were no adverse events associated with the intervention, and implementation of the procedures was feasible in the military and veteran hospital settings
Mackie et al., 2017 [102]	Staff and patient experience	• Semi-structured interview • Written participants exit questionnaire • Patient Health Questionnaire -9 (PHQ-9)	Seven men were enrolled in the study, and six completed the qualitative interviews. The two main themes identified were of trust and connection. Participants attended 85% of their appointments
Madan et al., 2015 [98]	Depression, suicide ideation and behaviour	• Patient Health Questionnaire -9 (PHQ-9) • Columbia Suicide Severity Rating Scale (CSSR-S)	At admission, 59.0% of patients endorsed suicidality on at least 1 of the suicide alert critical items. Patients endorsed critical item 1 (from the PHQ) most frequently and more often than any of critical items 2 to 6 from the CSSR-S. Patients who endorse more items may be experiencing more severe suicidality
Marasinghe et al., 2012 [79]	Suicide ideation, depression, social support, alcohol use, and drug check	• Beck Scale for Suicidal Ideation • Beck depression inventory • Medical outcomes study social support survey • Alcohol use disorders identification test • Drug check problem list	There were no significant differences between the groups at baseline. Intention-to-treat analyses showed that average scores for both conditions improved on all outcome measures
McManamaO'Brien et al., 2017 [125]	Patients and parent experience related to usability, and satisfaction	• System usability scale • Open ended survey questions	Results demonstrated acceptability and usability, suggesting the utility of technological interventions, such as Crisis Care, as an adjunct to treatment for suicidal adolescents and their parents following discharge from acute care settings
Melvin et al., 2019 [126]	Suicide ideation, coping, feasibility	• App usage data • App feedback survey (closed ended and open-ended) • Columbia Suicide Severity Rating Scale • Suicide related coping scale • Suicide resilience inventory-25 • Coping strategy usage questionnaire	A vast majority of participants used the app to view and edit their safety plans and reported that the app was easy to use. A reduction was observed in participant severity and intensity of suicide ideation, and suicide-related coping increased significantly. No significant changes were observed in suicide resilience
Morthorst et al., 2021[127]	Feasibility, clinical outcomes including NSSI, quality of life, sick days	• Phone interviews • Completion of follow-up, compliance (completion of modules) • deliberate self-harm inventory – youth version • Health-related quality of life questionnaire (kidscreen-10) • Depression anxiety stress scale • Number of sick days • Difficulties in emotion regulation scale–16 item version • Borderline symptom list • Columbia Suicide Severity Rating Scale • The coping with children’s negative emotions scale • The coping with children's negative emotions scale adolescent • Negative effects questionnaire • Strengths and difficulties questionnaire • Working alliance inventory, short version	Not Applicable—protocol
Mousavi 2014 [80]	Suicide attempts, suicide ideation, hope of life, compliance of treatment	• Phone calls (questionnaires)	The only suicide attempt case in the intervention group occurred in the 4th month after discharge, and in the control group there was one case after the 1st month, 2 cases after the 2nd month and one case after the 4th month. After discharge during the 6 month follow up, one patient in the intervention group and 4 patients in the control group had attempted suicide, no significant difference of suicide reattempt has been found between two groups. By the end of the study period, 28 patients in the control group and 14 patients in the intervention group had suicidal thoughts. 19 patients in the control group and 50 patients in the intervention group had increase in hope. There was no significant difference for the compliance to treatments after 6 months of follow up
Muscara et al., 2020 [128]	Feasibility, acceptability, suicide resilience and self-harm	• App log ins and use data • Self-report questionnaire • Suicide resilience inventory-25 measure	Eight participants felt that the apps would not keep them safe when in crisis, with nine and seven participants reported that BeyondNow and BlueIce, respectively, did not help them to manage their symptoms in crisis. Most participants rated both apps positively regarding ease of use, and a small majority reported that they would recommend both apps and were satisfied with the apps. Most participants did not believe that they would use the apps in the future. A significant improvement was found on the Emotional Stability Scale
Nuij et al., 2018 [129]	Feasibility, level of explorative power of the model, suicide behaviour	• System usability scale • Client satisfaction questionnaire 8 • Survey comprised of scale and questionnaires operationalised within the Integrated Motivational-Volitional model	Not Applicable—protocol
O'Keefe et al., 2019 [81]	Suicide ideation, resilience, depression, anxiety, impulsivity, self-efficacy, communal mastery, self-esteem, substance use	• Suicide Ideation Questionnaire • Resiliency scales for children and adolescents • Centers for epidemiologic studies depression scale revised • Children’s hope scale • Alcohol, smoking and substance involvement screening test • UPPS impulsive behavior scale • Multicultural mastery scale • Voices of Indian teens cultural issues and interest • Rosenberg self-esteem scale • Index of local indicators of well-being • PROMIS pediatric anxiety short form	Not Applicable—protocol
O'Toole et al., 2019 [82]	Suicide risk, depression, patient perception of the app	• Suicide Status Form (SSF) II–R • Major Depression Inventory (MDI) • Total app activity • Unsafe of methods library	A significant main effect of time on SSF was found across the whole intervention period, where self-reported suicide risk decreased. Concerning MDI, the main effect of time across the whole intervention period was significant, showing a large decrease across groups in depressive symptoms. Concerning the participants who had used the mobile app measured as any type of clicks (N = 50), the total number of clicks was not significant at either post-treatment. The total number of methods used was not significantly associated with the effect
O’Connor 2019 [135]	Feasibility, acceptability, intervention adherence, suicide severity, coping	• Columbia Suicide Severity Rating Scale • The entrapment scale • The interpersonal needs questionnaire • The ENRICHD social support instrument • The suicide-related coping scale • Semi-structured interview and focus group • NHS clinical databases	Not Applicable—protocol
Olsen et al., 2021 [130]	Feasibility, NSSI, quality of life, depression, anxiety, and stress	• Deliberate self-harm inventory–youth version • Kidscreen-10 • Depression anxiety stress scale • Proportion of sick days during the last month • Difficulties in emotion regulation scale • Borderline symptom list • Columbia Suicide Severity Rating Scale • Coping with children’s negative emotions scale • Negative effects questionnaire	Not Applicable—protocol
Owens and Charles 2016 [131]	Feasibility, clinician and patient experience	• Interview	Clinicians all understood the purpose of the intervention and recognised that it could be valuable in the management of self-harm and other problem behaviours, but heavy workloads, high stress levels and possibly some technophobia contributed to a perception that too much effort was required to master it and incorporate it into their practice
Canady 2018 [104]	Suicide risk	• Columbia Suicide Severity Rating Scale • Clinical practice screener-recent	In the ED, 6.3 percent of the screens were positive, as were 1.6 percent in the inpatient units, and 2.1 percent in the outpatient clinics
Pickett et al., 2021[132]	Feasibility, rate of screening, suicide risk	• Ask suicide screening questions	Suicide screening increased from 1.0% to 76.5%. The novel use of a tablet-based universal suicide screening method was successfully implemented in a busy ED and designed to optimize disclosure and patient comfort, while preserving valuable provider/nursing time
Sayal et al., 2019 [133]	Depression, suicide severity, anxiety, hopelessness, and health utility	• Beck Depression Inventory-II • Personal health questionnaire – 9 • Beck hopelessness scale • Generalised Anxiety Disorder Assessment • Columbia Suicide Severity Rating Scale • Work and social adjustment scale • 5-level EuroQol 5-dimensional questionnaire • Interviews	Recruitment to RCTs of remotely delivered CBT for young people with depression and repeat self-harm is not feasible through recent presentations to clinicians in self-harm services. Offering remotely delivered PSCBT did not enhance the uptake of this intervention in participants aged 16–30 years with depression who had recently presented to medical services following self-harm
Seong et al., 2021 [99]	Successful case management rate	• Case management database of the hospital	The rate of patients who connected with their local psychiatric healthcare center showed a significant difference between the Mobile Messenger Counselling (MMC) and non-MMC groups. The use of mobile messengers for counseling self-harm or suicide attempters leads to higher case management success rates by increasing their likelihood of connecting to a local psychiatric healthcare center
Simon et al., 2016 [63](protocol) Simon 2022 [83]	Self-harm, mortality suicide attempt	• Electronic health record data • Death certificate • Insurance claim data	Risk of fatal or nonfatal self-harm over 18 months did not differ significantly between the care management and usual care groups but was significantly higher in the skills training group than in usual care
Stallard et al., 2016 [64] protocol Stallard et al., 2018 [84]	Depression, anxiety, suicide behaviour, safety, acceptability, and self-harm, usability, feasibility	• Mood and feelings questionnaire • Revised child anxiety and depression scale • Strengths and difficulties questionnaire • Rating questionnaires • Semi-structured interviews • Referral pathways	No safety issues were identified and there were no unintended negative effects on self-harm. Almost three-quarters of those who had recently self-harmed reported reductions in self-harm after using BlueIce for 12 weeks. There was a statistically significant mean difference on post use symptoms of depression and symptoms of anxiety, which was evident across all anxiety subscales. Ratings of app acceptability and usefulness were high
Stevens et al., 2019 [85]	Hospitalization, mortality	• Routinely collected data sources through New South Wales (NSW) health, other government agencies, and the centre for health record linkage	Not Applicable—protocol
Vaiva et al., 2006 [87]	Suicide reattempt, death, number and type of health care contact	• Telephone interviews • Electronic health record data • Emergency departments health records on all suicide attempts, deaths, or further suicide attempts	70% of participants in both intervention groups were successfully contacted by telephone. Six participants died. On an intention to treat basis, the three groups did not differ significantly for proportion with an adverse outcome. The number of participants contacted at one month who reattempted suicide was significantly lower than that of controls. For participants contacted at three months, the number who attempted further suicide was not significantly lower than that of controls. Participants in the intervention groups talked about their attempted suicide with their general practitioner more often than the controls
Vaiva et al., 2011 [65] (protocol) Vaiva et al., 2018 [86]	Suicide reattempt, adverse events such death by suicide	• Mini-international neuropsychiatric interview • Phone survey	After 6 months, 58 participants in the intervention group reattempted suicide compared with 77 in the control group. The difference between groups was not significant
Wright et al., 2021 [134]	Depression, suicidality, and patient experience	• Beck depression inventory-II • Questions/observations during sessions (general comments on iPad use)	Of the 40 patient participants, 25% selected one of the depressive symptoms or one of the suicide responses on the depression inventory, made comments or displayed depressive symptoms in the audio-visual group sessions, or wrote about issues that caused the professionals to be concerned about possible suicidal ideation. All the patients commented on the iPad delivery being easy com-pared with some other open-source methods they had used. Various types of supportive interactions were observed among the group participants, including affirmations, humor, and emotional and in-formational support

## Discussion

### Summary of evidence

This scoping review describes characteristics of ICT-based interventions for suicide prevention implemented in clinical settings. In this review, we identified 75 papers that described 70 studies and 66 ICTs. Overall, the review findings provide detailed characteristics of the existing ICTs for suicide prevention implemented in clinical settings. We also identified common strategies for implementing these ICTs, related barriers and facilitators, as well as reported measures and outcomes of the included ICTs. The findings offer insights into how to better support the implementation of ICTs and highlight the important role of collaborative initiatives in providing both technical and social support to facilitate implementation of ICTs in clinical settings.

### Characteristics of included studies

Most of the included studies were experimental designs and feasibility trials, and there were 18 protocols, indicating that many studies are currently underway. Despite the growing evidence in this field, we found a lack of qualitative evidence. This is a gap in the current literature, and future research should consider qualitative study designs to evaluate implementation and/or impact of ICT-based interventions for suicide prevention on patients, health care providers, and health systems. This is because clinical practice within hospitals is an example of a complex adaptive system [26, 27]. Evaluating and understanding implementation of ICTs in complex systems will benefit from using qualitative or mixed-methods designs because quantitative methods alone cannot capture the complexity inherent within the phenomenon nor can it unpack interplay of contextual characteristics that influence implementation and impact of ICTs. Efforts are needed to move beyond traditional effectiveness trials and better understand how and why innovations bring change in what context [136]. Qualitative research designs can facilitate benefits of unpacking contextual factors (e.g., barriers and facilitators) at multiple levels (e.g., individual, system) and answering complex questions [137] that are integral to moving ICTs forward. Moreover, qualitative methods alone or in mixed-methods designs can confirm, complement, or extend quantitative evaluation of effectiveness, providing explanatory knowledge [138].

Based on the paucity of TMFs identified in the include studies, future research should consider using TMFs to guide their study. Despite the clinical potential of using mental health apps, integrating these apps into routine practice is limited, partly attributable to a lack of theoretical foundations and rigour in research for implementation [23]. Future research can benefit from leveraging TMFs and qualitative and/or mixed methods designs to unpack the complexity and contribute to building a rich evidence base. Benefits of using established TMFs in research have been well documented. For example, TMFs can help researchers consider comprehensive list implementation outcomes [139–141]. Furthermore, TMFs can help researchers consider a complete list of determinants for implementation during the planning phase to maximize implementation success [139–141]. Implementation is a known determinant of intervention effectiveness [56], and as we continue to face challenges in moving ICTs beyond pilot trials, it is necessary to leverage TMFs to guide careful and purposeful implementation that accounts for the complex contexts in which ICTs are implemented [22]. This will ensure that implementation strategies are systematically selected to address barriers in the local context. However, it is difficult to know whether authors of the included studies in this review did not use TMFs or did not report TMFs. If it is a reporting issue, then researchers need to improve reporting on TMFs so we can learn how TMFs have been applied, build knowledge base, and modify TMFs as necessary.

### Implementation of ICTs in clinical settings

Thirty-one studies reported implementation strategies and 19 studies reported barriers and facilitator. Despite the general lack of reporting details, useful insights about implementation supports can be drawn. Of the reported studies, education and training were the most commonly reported implementation strategies for the ICTs. This is consistent with the current literature for implementation practice and knowledge translation [142, 143]. Educational meetings and training workshops are less costly and more accessible to support implementation than complex strategies requiring organizational-level change [144]. Therefore, educational meetings and training workshops could have been feasible options. However, barriers related to psychological capabilities were the least frequently coded category in the included studies. It is important to note that improving clinicians’ level of knowledge and skills does not always lead to observable practice changes leading to successful implementation of innovations [145]. Therefore, we recommend strategically considering diverse types of implementation strategies, other than education and training, to target both clinician- and external-level barriers for a given context. Secondly, collaborative initiatives were the next commonly reported strategy for implementation identified in this review. While partnership approaches such as co-design are common for innovation development, people often think that implementing what has been designed is the responsibility of others [146]. This is not true; researchers can co-create changes in the workflow to support implementation [147]. We encourage researchers to continue to leverage collaborative initiatives within their studies as they can foster important relationships between knowledge users and researchers. This will allow researchers to focus on real-world needs and facilitating implementation efforts [148, 149].

Researchers need to consider the complex contexts in which apps are being implemented [22]. As such, reporting details of implementation plans are strongly encouraged to advance our understanding of implementation processes and context. During implementation, the influence of context, such as barriers and facilitators, and interactions between them, are necessary to explain how or why certain outcomes are achieved, as well as variations in outcomes across studies [150, 151]. Furthermore, implementation is a known determinant of intervention effectiveness, and barriers can significantly reduce the effectiveness of an intervention [56]. Not knowing contextual influences may limit the generalizability of study findings to different settings. In response to the general lack of reporting details identified in this review, we encourage future studies to consider Proctor’s recommendations for specifying and reporting implementation strategies [152] and the Expert Recommendations for Implementing Change (ERIC) taxonomy for implementation strategies [153]. Furthermore, considering the iterative nature of the implementation process, any changes to original implementation plans are also encouraged to be reported. Future studies can consider the Framework for Reporting Adaptations and Modifications–Enhanced (FRAME) to guide the reporting of adaptations and modifications to the design or delivery of an intervention [154].

It has been reported that researchers are faced with challenges of selecting implementation strategies [155]. Furthermore, implementation strategies have often been mismatched to existing barriers [156, 157]. For example, a review of 20 quality improvement studies found that many studies utilized clinician-oriented (individual-level) strategies, such as education, to address organizational-level barriers [156]. Similarly, the current review identified that the three most reported categories of barriers were related to physical opportunity, social opportunity and reflective motivation, and examples included poor internet connection, busy clinical settings, lack of buy-in from and engagement with clinicians, and perceived uncertainties around ICTs. However, the most reported implementation strategies were education and training support, all of which cannot address the barriers stated above. This is an example of missed opportunities and an area for future research efforts.

Guided by the BCW, we can identify intervention options that can address the barriers identified in this review. To overcome physical opportunity, *Training, Enablement, Environment Restructuring*, or *Restriction* are recommended [49]. To overcome social opportunity, *Restriction, Environment Restructuring, Modelling,* or *Enablement* are suggested [49]. The use of evidenced-based theories like the BCW can improve the selection of implementation strategies and subsequent integration of ICTs in clinical settings [139, 141]. Additionally, clinical practice within health systems as well as human behaviour are complex; it is not individual factors that facilitate implementation of a new innovation, but the dynamic interaction between them [28, 158]. Nonetheless, the BCW accounts for interactions between both internal (i.e., *capability*, *motivation*) and external (i.e., *opportunity*) factors that influence behaviour change [49]. Use of behaviour change theories will not downgrade the complexity, but rather it can help researchers organize complex data in a comprehensive way that is also accessible to work with. As such, we recommend future studies to use TMFs to guide the selection of implementation strategies to overcome existing barriers.

Consistent with the current review findings, other external barriers associated with implementing ICTs are related to limited access to ICTs and internet, and digital literacy skills [159]. Despite the widespread use of mobile phones, a phenomenon called the ‘digital divide’ can occur due to social equity factors such as education, income, age, and urban/rural residence [160–162]. Digital divide refers to inequities in accessing and using ICTs as well as associated outcomes of using ICTs [162]. To prevent digital divide amplification and to avoid unintended harm, implementation efforts for new innovations must account for digital equity considerations [163]. However, very few included studies considered equity concerns and provided patients with ICT devices [74, 101, 114, 134], free data plans [111], or options for alternative ICTs (e.g., email instead of texts) as per patients’ preferences [71, 133]. In contrast, several studies made ownership of ICT devices as one of the inclusion criteria [61, 69, 78, 82, 85, 88, 102, 105, 111, 113, 120, 128, 129, 131, 135], and one study excluded participants who reported difficulty using a computer [117]. This is a critical area of future efforts for minimizing the digital divide. Van Dijk [164, 165], and Selwyn [166] recommend addressing the digital divide through assessing patient ICT access, use, competence, and reasons for divided outcomes. As many ICTs are rapidly being adopted and implemented for suicide prevention, this review identified a lack of attention to equity-related considerations in the current literature. This highlights a critical direction for future research, as efforts are needed to prevent digital divide amplification and avoid unintended harm while advancing ICT use.

### Reported measures and outcomes

We identified that studies of ICT-based interventions for suicide prevention reported implementation outcomes and/or interventions’ impact on patients, clinicians, and/or health systems. Most studies reported patient-level outcomes, such as suicide risk and behaviours, and implementation outcomes of feasibility. However, no studies reported long-term outcomes of implementation such as sustainability. This is a gap in the current literature, and future research should consider assessing long-term outcomes, or at least should consider sustainability potential beyond feasibility. The end goal of implementing new innovations in clinical settings is routinization, achieving seamless integration of ICT use in routine clinical flow [167]. Despite the promising clinical benefits of ICTs for suicide prevention, clinical integration remains limited [22–24]. This problem is consistent across ICTs in general. It has been repeatedly reported that ICTs are not fully implemented, not moving beyond pilot trials, or being abandoned [25, 168]. To move beyond initial adoption of useful ICTs, we encourage future research to consider sustainability outcomes early on. Proctor’s Implementation Outcomes Framework [56] and the Reach, Effectiveness, Adoption, Implementation Maintenance (RE-AIM) [169] are example tools to guide outcome selections related to implementation and sustainability of interventions. Several studies included in this review measured both intervention outcomes and implementation outcomes in one study [67, 76, 80, 84, 89, 91, 101, 106, 114, 115, 117, 118, 120, 122, 124, 126–132, 135]. Similarly, future research can benefit from leveraging effectiveness-implementation hybrid designs that have a dual focus of evaluating intervention effectiveness and implementation outcomes simultaneously [170, 171]. Hybrid designs are encouraged to move interventions to the real-world more rapidly because the traditional research approach of keeping efficacy, effectiveness, and implementation research separate and sequential slows down the process and overlooks complex contexts inherent within [170, 171].

## Limitations

Several limitations may affect the interpretation and use of our review findings. Many papers lacked detail on the barriers and facilitator to implementation, which made challenging to categorize them into the three overarching domains of *capability*, *opportunity*, and *motivation*. We conducted directed content analysis of the barriers and facilitators, and we report the frequency counts of these barriers and facilitators. However, this may not be a complete list of barrier and facilitators to implementation. Additionally, the categories within the COM-B and TDF are not mutually exclusive; many barriers and facilitators interact with each other, and this is one of the underlining assumptions of the human behaviour [49].

Secondly, our search strategy was limited to papers published in English. This may partly explain our finding that most studies originated from North America and Europe. As shown in the Fig. 1, we excluded eight papers written in non-English languages. We also acknowledge that our search strategies may not have captured studies conducted in low and middle-income countries. As a result, this review does not reflect evidence of ICTs for suicide prevention written in non-English languages or low- and middle-income countries, possibly resulting in underrepresentation and/or underreporting of the authorship and the amount of literature.

Third, we did not include ICT-based interventions in non-clinical settings such as schools. There are many other ICT-based interventions for suicide prevention that exist beyond what is included in this review. Lastly, despite our comprehensive search strategy, which included varied terms to describe ICTs, it is possible that relevant literature was not captured. To mitigate this limitation, we used Google search as a complementary to locate additional studies that our search strategy might have missed. We believe that our final search strategies were sensitive enough to provide full coverage of relevant literature because many papers identified during the second step of Google search were already captured by our main database searches. It is also important to recognize the inherent limitation of Google searches related to reproducibility of results [172]. A researcher from a different country may receive different results with the same steps, which is why Google search was complementary to full search strategies and not used alone.

## Conclusions

This scoping review provides a comprehensive overview of published literature on the ICTs for suicide prevention implemented in clinical settings. The findings revealed the most common types of ICTs for suicide prevention, including apps, text messages, and telemedicine. These ICTs were commonly used as a targeted strategy for suicide prevention and served multiple functions, including suicide screening and assessment, safety planning, and post-discharge follow-up care. Additionally, the findings revealed that the most common strategies for implementing these ICTs included education, training, and collaborative initiatives. However, barriers collectively influenced clinicians’ capability, opportunity, and motivation to implement ICTs for suicide prevention. Therefore, implementation strategies must be tailored and multi-faceted to target specific barriers in a given context in order to facilitate implementation efforts for ICTs in clinical settings. Along with the lack of qualitative evidence in this field, the lack of reporting of implementation strategies and related barriers and facilitators was an evident gap in this body of literature, highlighting the need for more explorative research and a call for better reporting. Additionally, the lack of theoretical frameworks identified in included studies encourages the use of established TMFs to guide future work. Lastly, the absence of sustainability outcomes and digital equity considerations identified in the current literature highlights a critical direction for future research.

## Supplementary Information

**Additional file 1.** 

**Additional file 2.** 

**Additional file 3.** 

**Additional file 4.**

## Data Availability

All data generated or analysed during this study are included in this published article [and its Additional files].

## Abbreviations

ICT: Information and communication technology
BCW: Behaviour change wheel
COM-B: Capability opportunity motivation – behaviour
TDF: Theoretical domains framework
JBI: Joanna Briggs Institute
TMF: Theory, model, framework
