# Supplementary material for: Information and communication technology-based interventions for suicide prevention implemented in clinical settings: a scoping review
Source: BMC Health Serv Res. 2023 Mar 23;23:281. doi: 10.1186/s12913-023-09254-5 (PMC10037806; doi:10.1186/s12913-023-09254-5)
Supplement: Supplementary file 1 — Additional file 1. [file 12913_2023_9254_MOESM1_ESM.docx]

Additional File 1. Search strategies

Date searched: August 17-20, 2021

Ovid MEDLINE(R) <1946 to August Week 2 2021>

| Line | Query | Records Retrieved |
| --- | --- | --- |
| 1 | information technology/ | 527 |
| 2 | software/ | 115767 |
| 3 | mobile applications/ | 8322 |
| 4 | programming languages/ | 4242 |
| 5 | software design/ | 6050 |
| 6 | user-centered design/ | 56 |
| 7 | user-computer interface/ | 38392 |
| 8 | web browser/ | 1268 |
| 9 | word processing/ | 588 |
| 10 | exp electronics/ | 39340 |
| 11 | digital technology/ | 235 |
| 12 | exp computer simulation/ | 259171 |
| 13 | virtual reality/ | 3179 |
| 14 | video games/ | 6173 |
| 15 | electronic mail/ | 2802 |
| 16 | cell phone/ or smartphone/ | 15446 |
| 17 | text messaging/ | 3615 |
| 18 | wireless technology/ | 3917 |
| 19 | algorithms/ | 271665 |
| 20 | exp artificial intelligence/ | 119944 |
| 21 | exp expert systems/ | 3435 |
| 22 | exp fuzzy logic/ | 4765 |
| 23 | exp knowledge bases/ | 10548 |
| 24 | exp machine learning/ | 31030 |
| 25 | exp natural language processing/ | 4874 |
| 26 | exp neural networks, computer/ | 36240 |
| 27 | social media/ | 10877 |
| 28 | exp internet/ | 86669 |
| 29 | ((mobile or online or web or internet) adj3 (application* or app or apps or interface* or platform* or program* or tech* or intervention*)).tw,kf. | 31266 |
| 30 | (smartphone* or smart phone* or tablet or cellphone* or cell phone* or mobile phone* or iphone* or android*).tw,kf. | 44635 |
| 31 | Text messag*.tw,kf. | 3680 |
| 32 | (e-health or ehealth or m-health or mhealth).tw,kf. | 9636 |
| 33 | (social media* or social network* site* or social network* website* or facebook* or instagram* or snapchat* or Linkedin* or Weibo* or Whatsapp* or Whats app* or Telegram* or WeChat* or We Chat* or tiktok*).tw,kf. | 14451 |
| 34 | ((twitter* or tweet*) not tweetable abstract).tw,kf. | 3251 |
| 35 | (forum* adj3 (internet or web* or chat*)).tw,kf. | 543 |
| 36 | PatientsLikeMe.tw,kf. | 50 |
| 37 | wiki*.tw,kf. | 1181 |
| 38 | algorithm*.tw,kf. | 204873 |
| 39 | (artificial intelligence or "AI" or "A.I.").tw,kf. | 31600 |
| 40 | computational intelligence.tw,kf. | 241 |
| 41 | (machine learning or deep learning or natural language processing).tw,kf. | 38496 |
| 42 | (information adj3 technolog*).tw,kf. | 16669 |
| 43 | (communication* adj3 technolog*).tw,kf. | 4942 |
| 44 | programming language*.tw,kf. | 1319 |
| 45 | software design*.tw,kf. | 765 |
| 46 | user-centered design*.tw,kf. | 592 |
| 47 | user-computer interface*.tw,kf. | 150 |
| 48 | (web browser* or word process*).tw,kf. | 2428 |
| 49 | digital technolog*.tw,kf. | 1859 |
| 50 | ((computer* or digital* or virtual*) adj3 simulat*).tw,kf. | 25611 |
| 51 | virtual realit*.tw,kf. | 9127 |
| 52 | video gam*.tw,kf. | 3168 |
| 53 | wireless technolog*.tw,kf. | 452 |
| 54 | expert system*.tw,kf. | 2757 |
| 55 | fuzzy logic*.tw,kf. | 1481 |
| 56 | neural network*.tw,kf. | 41064 |
| 57 | or/1-56 | 955892 |
| 58 | suicide/ or suicidal ideation/ or suicide, attempted/ or suicide, completed/ | 61508 |
| 59 | (suicid* adj3 prevent*).tw,kf. | 6874 |
| 60 | (suicid* adj3 (attempt* or commit* or complet* or die* or dead or ideation* or thought* or plan* or consider* or contemplat* or behavio?r* or method*)).tw,kf. | 35196 |
| 61 | suicidal*.tw,kf. | 29578 |
| 62 | self-injurious behavior/ or self mutilation/ | 11627 |
| 63 | (selfharm* or self-harm or selfinjur* or self-injur* or selfinflict* or self-inflict* or self-mutilat* or selfmutilat* or selfpoison* or self-poison* or automutilat*).tw,kf. | 14027 |
| 64 | ((fatal* or lethal* or intentional* or deliberate*) adj2 (dose or doses or dosing or overdos* or self-administ* or selfadminist*)).tw,kf. | 12822 |
| 65 | or/58-64 | 96674 |
| 66 | practice patterns, dentists'/ or practice patterns, nurses'/ or practice patterns, physicians'/ | 68658 |
| 67 | "delivery of health care"/ | 99086 |
| 68 | "delivery of health care, integrated"/ | 13511 |
| 69 | patient care management/ | 4587 |
| 70 | health services accessibility/ | 79932 |
| 71 | managed care programs/ | 24337 |
| 72 | telemedicine/ | 29469 |
| 73 | exp Health Facilities/ | 825288 |
| 74 | (clinical* adj3 (practic* or practis* or application* or care)).tw,kf. | 308798 |
| 75 | ((health or healthcare or medical or psychiatr* or mental* or rehab* or treatment* or inpatient* or outpatient* or walk-in or drop-in) adj3 (hospital* or institut* or setting* or environment* or clinic* or centre* or center* or facility or facilities or ward* or unit* or office* or program* or service* or intervention*)).tw,kf,hw. | 1349386 |
| 76 | (clinical adj3 (hospital* or institut* or setting* or environment* or centre* or center* or facility or facilities or ward* or unit* or office* or program* or service* or intervention*)).ti,ab,kf,hw. | 152863 |
| 77 | (care team* or healthcare team* or health team*).tw,kf. | 16811 |
| 78 | ((health* or healthcare or medical* or mental* or psychiatr* or inpatient* or outpatient*) adj3 (session* or appointment*)).tw,kf. | 4910 |
| 79 | ((nurse* or nursing) adj3 (hospital* or institut* or setting* or environment* or clinic* or centre* or center* or facility or facilities or ward* or unit* or office* or program* or service* or intervention* or care)).ti,ab,kf,hw. | 205395 |
| 80 | medical home*.tw,kf. | 3174 |
| 81 | hospital*.tw,kf. | 1211999 |
| 82 | (emergency adj3 (department* or ward* or unit* or room* or service* or medicine or center* or centre* or clinic or clinics or hospital* or care or visit* or patient*)).tw,kf. | 151142 |
| 83 | (casualty adj1 (department* or ward* or unit* or room* or service* or medicine or center* or centre* or clinic or clinics or hospital* or visit* or patient*)).tw,kf. | 890 |
| 84 | "a&e".tw,kf. | 21392 |
| 85 | ("ER" or "E.R.").ti. | 9037 |
| 86 | (telemental* or tele mental* or teletherap* or telepsych* or telemedic* or telehealth* or teleconferenc* or tele-psychotherap* or tele-psychiatr* or tele-medic* or tele-health* or tele-conferenc*).tw,kf. | 21131 |
| 87 | ((health* or healthcare or medical* or mental* or psychiatr*) adj3 (profession* or work* or practitioner* or provider* or clinician* or servic*)).tw,kf. | 440894 |
| 88 | (physician* or doctor* or primary care or general practitioner* or nurse practitioner*).tw,kf. | 574724 |
| 89 | (psychiatrist* or psychologist*).tw,kf. | 34650 |
| 90 | ((health* or medic* or clinic*) adj3 (aide* or assistant* or technician* or navigator*)).tw,kf. | 7397 |
| 91 | (peer* adj3 (worker* or coach* or navigator*)).tw,kf. | 807 |
| 92 | (digital adj3 (coach* or navigator*)).tw,kf. | 32 |
| 93 | ((regulated or unregulated) adj3 (provider* or professional* or worker*)).tw,kf. | 187 |
| 94 | (treatment* or intervention*).ti,hw. | 2174461 |
| 95 | (allerg* or immunolog* or anesthesiolog* or dermatolog* or radiolog* or emergency medicine or family medicine or internal medicine or internist* or neurology* or obstetric* or gynecolog* or ophthalmolog* or pathology or pathologist* or pediatric* or paediatric* or oncolog* or surgeon* or surgical* or surgery or urolog*).tw,kf. | 3277960 |
| 96 | (social work* or occupational therap* or allied health* or pharmacy or pharmacist* or physiotherap* or dentist* or dental* or audiolog* or speech patholog* or language patholog* or chiropod* or podiatr* or chiropract* or dentur* or dietician* or dietetic* or homeopath* or naturopath* or kinesiolog* or massage therap* or midwif* or midwiv* or optician* or optometr* or psychotherap* or psycho-therap* or respiratory therap* or chinese medicine or acupunctur* or (laborator* adj3 (technolog* or technician*)) or ((radiation or radiolog*) adj3 (technolog* or technician*))).tw,kf. | 540714 |
| 97 | or/66-96 | 7656426 |
| 98 | 57 and 65 and 97 | 1087 |

Ovid MEDLINE(R) Epub Ahead of Print <August 19, 2021>; Ovid MEDLINE(R) In-Process & In-Data-Review Citations <1946 to August 19, 2021>

| Line | Query | Records Retrieved |
| --- | --- | --- |
| 1 | programming language*.tw,kf. | 94 |
| 2 | software design*.tw,kf. | 36 |
| 3 | user-centered design*.tw,kf. | 68 |
| 4 | user-computer interface*.tw,kf. | 10 |
| 5 | (web browser* or word process*).tw,kf. | 83 |
| 6 | digital technolog*.tw,kf. | 322 |
| 7 | ((computer* or digital* or virtual*) adj3 simulat*).tw,kf. | 785 |
| 8 | virtual realit*.tw,kf. | 949 |
| 9 | video gam*.tw,kf. | 195 |
| 10 | wireless technolog*.tw,kf. | 27 |
| 11 | expert system*.tw,kf. | 47 |
| 12 | fuzzy logic*.tw,kf. | 91 |
| 13 | neural network*.tw,kf. | 5442 |
| 14 | ((mobile or online or web or internet) adj3 (application* or app or apps or interface* or platform* or program* or tech* or intervention*)).tw,kf. | 2864 |
| 15 | (smartphone* or smart phone* or tablet* or cellphone* or cell phone* or mobile phone* or iphone* or android*).tw,kf. | 3506 |
| 16 | Text messag*.tw,kf. | 304 |
| 17 | (e-health or ehealth or m-health or mhealth).tw,kf. | 956 |
| 18 | (social media* or social network* site* or social network* website* or facebook* or instagram* or snapchat* or Linkedin* or Weibo* or Whatsapp* or Whats app* or Telegram* or WeChat* or We Chat* or tiktok*).tw,kf. | 2587 |
| 19 | ((twitter* or tweet*) not tweetable abstract).tw,kf. | 499 |
| 20 | (forum* adj3 (internet or web* or chat*)).tw,kf. | 35 |
| 21 | PatientsLikeMe.tw,kf. | 1 |
| 22 | wiki*.tw,kf. | 68 |
| 23 | algorithm*.tw,kf. | 13683 |
| 24 | (artificial intelligence or "AI" or "A.I.").tw,kf. | 3647 |
| 25 | computational intelligence.tw,kf. | 13 |
| 26 | (machine learning or deep learning or natural language processing).tw,kf. | 9366 |
| 27 | (information adj3 technolog*).tw,kf. | 997 |
| 28 | (communication* adj3 technolog*).tw,kf. | 322 |
| 29 | or/1-28 | 35697 |
| 30 | suicide/ or suicidal ideation/ or suicide, attempted/ or suicide, completed/ | 0 |
| 31 | (suicid* adj3 prevent*).tw,kf. | 602 |
| 32 | (suicid* adj3 (attempt* or commit* or complet* or die* or dead or ideation* or thought* or plan* or consider* or contemplat* or behavio?r* or method*)).tw,kf. | 2252 |
| 33 | suicidal*.tw,kf. | 2105 |
| 34 | self-injurious behavior/ or self mutilation/ | 0 |
| 35 | (selfharm* or self-harm or selfinjur* or self-injur* or selfinflict* or self-inflict* or self-mutilat* or selfmutilat* or selfpoison* or self-poison* or automutilat*).tw,kf. | 897 |
| 36 | ((fatal* or lethal* or intentional* or deliberate*) adj2 (dose or doses or dosing or overdos* or self-administ* or selfadminist*)).tw,kf. | 337 |
| 37 | or/30-36 | 3686 |
| 38 | (clinical* adj3 (practice* or practise* or application* or care)).tw,kf. | 18716 |
| 39 | ((health or healthcare or medical or psychiatr* or mental* or rehab* or treatment* or inpatient* or outpatient* or walk-in or drop-in) adj3 (hospital* or institut* or setting* or environment* or clinic* or centre* or center* or facility or facilities or ward* or unit* or office* or program* or service* or intervention*)).tw,kf,hw. | 48012 |
| 40 | (clinical adj3 (hospital* or institut* or setting* or environment* or centre* or center* or facility or facilities or ward* or unit* or office* or program* or service* or intervention*)).ti,ab,kf,hw. | 8555 |
| 41 | (care team* or healthcare team* or health team*).tw,kf. | 1251 |
| 42 | ((health* or healthcare or medical* or mental* or psychiatr* or inpatient* or outpatient*) adj3 (session* or appointment*)).tw,kf. | 342 |
| 43 | ((nurse* or nursing) adj3 (hospital* or institut* or setting* or environment* or clinic* or centre* or center* or facility or facilities or ward* or unit* or office* or program* or service* or intervention* or care)).ti,ab,kf,hw. | 4881 |
| 44 | medical home*.tw,kf. | 114 |
| 45 | hospital*.tw,kf. | 53598 |
| 46 | (emergency adj3 (department* or ward* or unit* or room* or service* or medicine or center* or centre* or clinic or clinics or hospital* or care or visit* or patient*)).tw,kf. | 9035 |
| 47 | (casualty adj1 (department* or ward* or unit* or room* or service* or medicine or center* or centre* or clinic or clinics or hospital* or visit* or patient*)).tw,kf. | 9 |
| 48 | "a&e".tw,kf. | 1248 |
| 49 | ("ER" or "E.R.").ti. | 404 |
| 50 | (telemental* or tele mental* or teletherap* or telepsych* or telemedic* or telehealth* or teleconferenc* or tele-psychotherap* or tele-psychiatr* or tele-medic* or tele-health* or tele-conferenc*).tw,kf. | 2556 |
| 51 | ((health* or healthcare or medical* or mental* or psychiatr*) adj3 (profession* or work* or practitioner* or provider* or clinician* or servic*)).tw,kf. | 26924 |
| 52 | (physician* or doctor* or primary care or general practitioner* or nurse practitioner*).tw,kf. | 21426 |
| 53 | (psychiatrist* or psychologist*).tw,kf. | 1433 |
| 54 | ((health* or medic* or clinic*) adj3 (aide* or assistant* or technician* or navigator*)).tw,kf. | 405 |
| 55 | (peer* adj3 (worker* or coach* or navigator*)).tw,kf. | 85 |
| 56 | (digital adj3 (coach* or navigator*)).tw,kf. | 4 |
| 57 | ((regulated or unregulated) adj3 (provider* or professional* or worker*)).tw,kf. | 14 |
| 58 | (treatment* or intervention*).ti,hw. | 36663 |
| 59 | (allerg* or immunolog* or anesthesiolog* or dermatolog* or radiolog* or emergency medicine or family medicine or internal medicine or internist* or neurology* or obstetric* or gynecolog* or ophthalmolog* or pathology or pathologist* or pediatric* or paediatric* or oncolog* or surgeon* or surgical* or surgery or urolog*).tw,kf. | 124166 |
| 60 | (social work* or occupational therap* or allied health* or pharmacy or pharmacist* or physiotherap* or dentist* or dental* or audiolog* or speech patholog* or language patholog* or chiropod* or podiatr* or chiropract* or dentur* or dietician* or dietetic* or homeopath* or naturopath* or kinesiolog* or massage therap* or midwif* or midwiv* or optician* or optometr* or psychotherap* or psycho-therap* or respiratory therap* or chinese medicine or acupunctur* or (laborator* adj3 (technolog* or technician*)) or ((radiation or radiolog*) adj3 (technolog* or technician*))).tw,kf. | 18263 |
| 61 | or/38-60 | 251401 |
| 62 | 29 and 37 and 61 | 89 |

Embase Classic+Embase <1947 to 2021 August 17>

| Line | Query | Records Retrieved |
| --- | --- | --- |
| 1 | information technology/ | 11775 |
| 2 | *software/ | 11568 |
| 3 | exp mobile application/ | 16690 |
| 4 | computer language/ | 5679 |
| 5 | computer interface/ | 33285 |
| 6 | exp information technology device/ | 202701 |
| 7 | electronics/ | 31193 |
| 8 | computer simulation/ | 126101 |
| 9 | virtual reality/ | 20033 |
| 10 | video game/ | 4404 |
| 11 | e-mail/ | 25458 |
| 12 | mobile phone/ or smartphone/ | 35296 |
| 13 | text messaging/ | 5979 |
| 14 | wireless communication/ | 6048 |
| 15 | exp algorithm/ | 446717 |
| 16 | exp artificial intelligence/ | 50917 |
| 17 | expert system/ | 5528 |
| 18 | fuzzy logic/ | 4283 |
| 19 | knowledge base/ | 6162 |
| 20 | exp machine learning/ | 264992 |
| 21 | natural language processing/ | 6656 |
| 22 | artificial neural network/ | 40038 |
| 23 | social media/ | 28886 |
| 24 | web-based intervention/ | 1137 |
| 25 | ((mobile or online or web or internet) adj3 (application* or app or apps or interface* or platform* or program* or tech* or intervention*)).tw,kw. | 55795 |
| 26 | (smartphone* or smart phone* or tablet or cellphone* or cell phone* or mobile phone* or iphone* or android*).tw,kw. | 91229 |
| 27 | Text messag*.tw,kw. | 6455 |
| 28 | (e-health or ehealth or m-health or mhealth).tw,kw. | 15092 |
| 29 | (social media* or social network* site* or social network* website* or facebook* or instagram* or snapchat* or Linkedin* or Weibo* or Whatsapp* or Whats app* or Telegram* or WeChat* or We Chat* or tiktok*).tw,kw. | 29213 |
| 30 | ((twitter* or tweet*) not tweetable abstract).tw,kw. | 6367 |
| 31 | (forum* adj3 (internet or web* or chat*)).tw,kw. | 1053 |
| 32 | PatientsLikeMe.tw,kw. | 150 |
| 33 | wiki*.tw,kw. | 2059 |
| 34 | algorithm*.tw,kw. | 368915 |
| 35 | (artificial intelligence or "AI" or "A.I.").tw,kw. | 57927 |
| 36 | computational intelligence.tw,kw. | 474 |
| 37 | (machine learning or deep learning or natural language processing).tw,kw. | 80336 |
| 38 | (information adj3 technolog*).tw,kw. | 25766 |
| 39 | (communication* adj3 technolog*).tw,kw. | 8024 |
| 40 | programming language*.tw,kw. | 2362 |
| 41 | software design*.tw,kw. | 1440 |
| 42 | user-centered design*.tw,kw. | 922 |
| 43 | user-computer interface*.tw,kw. | 305 |
| 44 | (web browser* or word process*).tw,kw. | 3418 |
| 45 | digital technolog*.tw,kw. | 3367 |
| 46 | ((computer* or digital* or virtual*) adj3 simulat*).tw,kw. | 38381 |
| 47 | virtual realit*.tw,kw. | 16226 |
| 48 | video gam*.tw,kw. | 5220 |
| 49 | wireless technolog*.tw,kw. | 837 |
| 50 | expert system*.tw,kw. | 4505 |
| 51 | fuzzy logic*.tw,kw. | 2924 |
| 52 | neural network*.tw,kw. | 79019 |
| 53 | or/1-52 | 1356874 |
| 54 | suicide/ | 63759 |
| 55 | suicidal behavior/ or self immolation/ or self poisoning/ or suicidal ideation/ or suicide/ or suicide attempt/ | 115873 |
| 56 | (suicid* adj3 prevent*).tw,kw. | 9942 |
| 57 | (suicid* adj3 (attempt* or commit* or complet* or die* or dead or ideation* or thought* or plan* or consider* or contemplat* or behavio?r* or method*)).tw,kw. | 56281 |
| 58 | suicidal*.tw,kw. | 48028 |
| 59 | (selfharm* or self-harm or selfinjur* or self-injur* or selfinflict* or self-inflict* or self-mutilat* or selfmutilat* or selfpoison* or self-poison* or automutilat*).tw,kw. | 21945 |
| 60 | ((fatal* or lethal* or intentional* or deliberate*) adj2 (dose or doses or dosing or overdos* or self-administ* or selfadminist*)).tw,kw. | 20125 |
| 61 | or/54-60 | 159058 |
| 62 | clinical practice/ | 312102 |
| 63 | exp medical care/ | 1083333 |
| 64 | health care delivery/ or exp ambulatory care/ or exp community care/ or exp hospital care/ or exp institutional care/ or exp long term care/ or exp nursing care/ or exp primary health care/ or exp telehealth/ | 3382862 |
| 65 | exp patient care/ | 892376 |
| 66 | health care facility/ | 78213 |
| 67 | ambulance/ | 14977 |
| 68 | cancer center/ | 41935 |
| 69 | community mental health center/ | 3842 |
| 70 | health center/ | 37923 |
| 71 | hospice/ | 14283 |
| 72 | exp hospital/ | 1320600 |
| 73 | mental health center/ | 6261 |
| 74 | pain clinic/ | 3880 |
| 75 | rehabilitation center/ | 17580 |
| 76 | (clinical* adj3 (practic* or practis* or application* or care)).tw,kw. | 529818 |
| 77 | ((health or healthcare or medical or psychiatr* or mental* or rehab* or treatment* or inpatient* or outpatient* or walk-in or drop-in) adj3 (hospital* or institut* or setting* or environment* or clinic* or centre* or center* or facility or facilities or ward* or unit* or office* or program* or service* or intervention*)).tw,kw,hw. | 2123827 |
| 78 | (clinical adj3 (hospital* or institut* or setting* or environment* or centre* or center* or facility or facilities or ward* or unit* or office* or program* or service* or intervention*)).tw,kw,hw. | 278863 |
| 79 | (care team* or healthcare team* or health team*).tw,kw. | 31785 |
| 80 | ((health* or healthcare or medical* or mental* or psychiatr* or inpatient* or outpatient*) adj3 (session* or appointment*)).tw,kw. | 12057 |
| 81 | ((nurse* or nursing) adj3 (hospital* or institut* or setting* or environment* or clinic* or centre* or center* or facility or facilities or ward* or unit* or office* or program* or service* or intervention* or care)).tw,kw,hw. | 198583 |
| 82 | medical home*.tw,kw. | 5068 |
| 83 | hospital*.tw,kw. | 2232459 |
| 84 | (emergency adj3 (department* or ward* or unit* or room* or service* or medicine or center* or centre* or clinic or clinics or hospital* or care or visit* or patient*)).tw,kw. | 283048 |
| 85 | (casualty adj1 (department* or ward* or unit* or room* or service* or medicine or center* or centre* or clinic or clinics or hospital* or visit* or patient*)).tw,kw. | 1334 |
| 86 | "a&e".tw,kw. | 52262 |
| 87 | ("ER" or "E.R.").ti. | 16451 |
| 88 | (telemental* or tele mental* or teletherap* or telepsych* or telemedic* or telehealth* or teleconferenc* or tele-psychotherap* or tele-psychiatr* or tele-medic* or tele-health* or tele-conferenc*).tw,kw. | 36043 |
| 89 | ((health* or healthcare or medical* or mental* or psychiatr*) adj3 (profession* or work* or practitioner* or provider* or clinician* or servic*)).tw,kw. | 673625 |
| 90 | (physician* or doctor* or primary care or general practitioner* or nurse practitioner*).tw,kw. | 954239 |
| 91 | (psychiatrist* or psychologist*).tw,kw. | 68897 |
| 92 | ((health* or medic* or clinic*) adj3 (aide* or assistant* or technician* or navigator*)).tw,kw. | 12555 |
| 93 | (peer* adj3 (worker* or coach* or navigator*)).tw,kw. | 1405 |
| 94 | (digital adj3 (coach* or navigator*)).tw,kw. | 103 |
| 95 | ((regulated or unregulated) adj3 (provider* or professional* or worker*)).tw,kw. | 260 |
| 96 | (treatment* or intervention*).ti,hw. | 3773435 |
| 97 | (allerg* or immunolog* or anesthesiolog* or dermatolog* or radiolog* or emergency medicine or family medicine or internal medicine or internist* or neurology* or obstetric* or gynecolog* or ophthalmolog* or pathology or pathologist* or pediatric* or paediatric* or oncolog* or surgeon* or surgical* or surgery or urolog*).tw,kw. | 5521101 |
| 98 | (social work* or occupational therap* or allied health* or pharmacy or pharmacist* or physiotherap* or dentist* or dental* or audiolog* or speech patholog* or language patholog* or chiropod* or podiatr* or chiropract* or dentur* or dietician* or dietetic* or homeopath* or naturopath* or kinesiolog* or massage therap* or midwif* or midwiv* or optician* or optometr* or psychotherap* or psycho-therap* or respiratory therap* or chinese medicine or acupunctur* or (laborator* adj3 (technolog* or technician*)) or ((radiation or radiolog*) adj3 (technolog* or technician*))).tw,kw. | 828189 |
| 99 | or/62-98 | 13713876 |
| 100 | 53 and 61 and 99 | 2709 |
| 101 | limit 100 to (conference abstracts or medline) | 1080 |
| 102 | limit 100 to (books or chapter) | 2 |
| 103 | 100 not (101 or 102) | 1627 |

APA PsycInfo <1806 to August Week 2 2021>

| Line | Query | Records Retrieved |
| --- | --- | --- |
| 1 | exp "information and communication technology"/ | 184217 |
| 2 | exp computer software/ | 15030 |
| 3 | mobile applications/ | 1298 |
| 4 | exp computer programming/ | 2423 |
| 5 | exp human machine systems/ | 7889 |
| 6 | human computer interaction/ | 11395 |
| 7 | exp websites/ | 6117 |
| 8 | word processing/ | 1009 |
| 9 | exp electronic communication/ | 36429 |
| 10 | exp digital technology/ | 139615 |
| 11 | exp computer simulation/ | 18395 |
| 12 | exp Virtual Reality/ | 9724 |
| 13 | computer games/ | 8078 |
| 14 | mobile phones/ or smartphones/ | 6053 |
| 15 | text messaging/ | 1179 |
| 16 | wireless technologies/ | 571 |
| 17 | algorithms/ | 20552 |
| 18 | exp artificial intelligence/ | 23768 |
| 19 | exp expert systems/ | 9039 |
| 20 | fuzzy logic/ | 1662 |
| 21 | exp machine learning/ | 10971 |
| 22 | natural language processing/ | 614 |
| 23 | neural networks/ or exp artificial neural networks/ | 19894 |
| 24 | exp social media/ | 17008 |
| 25 | internet/ | 29784 |
| 26 | ((mobile or online or web or internet) adj3 (application* or app or apps or interface* or platform* or program* or tech* or intervention*)).tw,id. | 21268 |
| 27 | (smartphone* or smart phone* or tablet or cellphone* or cell phone* or mobile phone* or iphone* or android*).tw,id. | 14121 |
| 28 | Text messag*.tw,id. | 2622 |
| 29 | (e-health or ehealth or m-health or mhealth).tw,id. | 3409 |
| 30 | (social media* or social network* site* or social network* website* or facebook* or instagram* or snapchat* or Linkedin* or Weibo* or Whatsapp* or Whats app* or Telegram* or WeChat* or We Chat* or tiktok*).tw,id. | 21406 |
| 31 | ((twitter* or tweet*) not tweetable abstract).tw,id. | 3663 |
| 32 | (forum* adj3 (internet or web* or chat*)).tw,id. | 721 |
| 33 | PatientsLikeMe.tw,id. | 23 |
| 34 | wiki*.tw,id. | 1294 |
| 35 | algorithm*.tw,id. | 35316 |
| 36 | (artificial intelligence or "AI" or "A.I.").tw,id. | 9362 |
| 37 | computational intelligence.tw,id. | 296 |
| 38 | (machine learning or deep learning or natural language processing).tw,id. | 10721 |
| 39 | (information adj3 technolog*).tw,id. | 15042 |
| 40 | (communication* adj3 technolog*).tw,id. | 8359 |
| 41 | programming language*.tw,id. | 773 |
| 42 | software design*.tw,id. | 596 |
| 43 | user-centered design*.tw,id. | 434 |
| 44 | user-computer interface*.tw,id. | 49 |
| 45 | (web browser* or word process*).tw,id. | 2749 |
| 46 | digital technolog*.tw,id. | 2668 |
| 47 | ((computer* or digital* or virtual*) adj3 simulat*).tw,id. | 7035 |
| 48 | virtual realit*.tw,id. | 7120 |
| 49 | video gam*.tw,id. | 6110 |
| 50 | wireless technolog*.tw,id. | 163 |
| 51 | expert system*.tw,id. | 1869 |
| 52 | fuzzy logic*.tw,id. | 860 |
| 53 | neural network*.tw,id. | 20095 |
| 54 | or/1-53 | 281886 |
| 55 | suicide/ or attempted suicide/ or suicidality/ | 36674 |
| 56 | exp self-injurious behavior/ or self-mutilation/ | 6600 |
| 57 | (suicid* adj3 prevent*).tw,id. | 8700 |
| 58 | (suicid* adj3 (attempt* or commit* or complet* or die* or dead or ideation* or thought* or plan* or consider* or contemplat* or behavio?r* or method*)).tw,id. | 41166 |
| 59 | suicidal*.tw,id. | 36558 |
| 60 | (selfharm* or self-harm or selfinjur* or self-injur* or selfinflict* or self-inflict* or self-mutilat* or selfmutilat* or selfpoison* or self-poison* or automutilat*).tw,id. | 14463 |
| 61 | ((fatal* or lethal* or intentional* or deliberate*) adj2 (dose or doses or dosing or overdos* or self-administ* or selfadminist*)).tw,id. | 954 |
| 62 | or/55-61 | 70098 |
| 63 | health care delivery/ | 21417 |
| 64 | exp clinical practice/ | 21821 |
| 65 | exp health care services/ | 221097 |
| 66 | exp telemedicine/ | 9954 |
| 67 | (clinical* adj3 (practic* or practis* or application* or care)).tw,id. | 77706 |
| 68 | ((health or healthcare or medical or psychiatr* or mental* or rehab* or treatment* or inpatient* or outpatient* or walk-in or drop-in) adj3 (hospital* or institut* or setting* or environment* or clinic* or centre* or center* or facility or facilities or ward* or unit* or office* or program* or service* or intervention*)).tw,id,hw. | 407722 |
| 69 | (clinical adj3 (hospital* or institut* or setting* or environment* or centre* or center* or facility or facilities or ward* or unit* or office* or program* or service* or intervention*)).tw,id,hw. | 43340 |
| 70 | (care team* or healthcare team* or health team*).tw,id. | 5974 |
| 71 | ((health* or healthcare or medical* or mental* or psychiatr* or inpatient* or outpatient*) adj3 (session* or appointment*)).tw,id. | 2492 |
| 72 | ((nurse* or nursing) adj3 (hospital* or institut* or setting* or environment* or clinic* or centre* or center* or facility or facilities or ward* or unit* or office* or program* or service* or intervention* or care)).tw,id,hw. | 37536 |
| 73 | medical home*.tw,id. | 1090 |
| 74 | hospital*.tw,id. | 166813 |
| 75 | (emergency adj3 (department* or ward* or unit* or room* or service* or medicine or center* or centre* or clinic or clinics or hospital* or care or visit* or patient*)).tw,id. | 19181 |
| 76 | (casualty adj1 (department* or ward* or unit* or room* or service* or medicine or center* or centre* or clinic or clinics or hospital* or visit* or patient*)).tw,id. | 68 |
| 77 | "a&e".tw,id. | 1818 |
| 78 | ("ER" or "E.R.").ti. | 321 |
| 79 | (telemental* or tele mental* or teletherap* or telepsych* or telemedic* or telehealth* or teleconferenc* or tele-psychotherap* or tele-psychiatr* or tele-medic* or tele-health* or tele-conferenc*).tw,id. | 5614 |
| 80 | ((health* or healthcare or medical* or mental* or psychiatr*) adj3 (profession* or work* or practitioner* or provider* or clinician* or servic*)).tw,id. | 215024 |
| 81 | (physician* or doctor* or primary care or general practitioner* or nurse practitioner*).tw,id. | 133422 |
| 82 | (psychiatrist* or psychologist*).tw,id. | 125713 |
| 83 | ((health* or medic* or clinic*) adj3 (aide* or assistant* or technician* or navigator*)).tw,id. | 1658 |
| 84 | (peer* adj3 (worker* or coach* or navigator*)).tw,id. | 1085 |
| 85 | (digital adj3 (coach* or navigator*)).tw,id. | 28 |
| 86 | ((regulated or unregulated) adj3 (provider* or professional* or worker*)).tw,id. | 117 |
| 87 | (treatment* or intervention*).ti,hw. | 434369 |
| 88 | (allerg* or immunolog* or anesthesiolog* or dermatolog* or radiolog* or emergency medicine or family medicine or internal medicine or internist* or neurology* or obstetric* or gynecolog* or ophthalmolog* or pathology or pathologist* or pediatric* or paediatric* or oncolog* or surgeon* or surgical* or surgery or urolog*).tw,id. | 185109 |
| 89 | (social work* or occupational therap* or allied health* or pharmacy or pharmacist* or physiotherap* or dentist* or dental* or audiolog* or speech patholog* or language patholog* or chiropod* or podiatr* or chiropract* or dentur* or dietician* or dietetic* or homeopath* or naturopath* or kinesiolog* or massage therap* or midwif* or midwiv* or optician* or optometr* or psychotherap* or psycho-therap* or respiratory therap* or chinese medicine or acupunctur* or (laborator* adj3 (technolog* or technician*)) or ((radiation or radiolog*) adj3 (technolog* or technician*))).tw,id. | 214781 |
| 90 | or/63-89 | 1312574 |
| 91 | 54 and 62 and 90 | 1099 |
| 92 | limit 91 to dissertation | 51 |
| 93 | limit 91 to chapter | 70 |
| 94 | 91 not (92 or 93) | 978 |

CINAHL Plus with Full Text – August 20, 2021

| Line | Query | Records Retrieved |
| --- | --- | --- |
| 1 | (MH "Information Technology") | 14,472 |
| 2 | (MM "Software+") | 19,541 |
| 3 | (MH "Mobile Applications") | 9,364 |
| 4 | (MM "Programming Languages") | 288 |
| 5 | (MM "Software Design") | 1,587 |
| 6 | (MM "User-Computer Interface+") | 4,392 |
| 7 | (MM "Web Browsers") | 199 |
| 8 | (MM "Word Processing") | 261 |
| 9 | (MH "Electronics") | 2,256 |
| 10 | (MH "Digital Technology+") | 4,210 |
| 11 | (MH "Computer Simulation+") | 24,118 |
| 12 | (MH "Virtual Reality+") | 5,904 |
| 13 | (MH "Video Games+") | 5,209 |
| 14 | (MH "Email") | 6,821 |
| 15 | (MH "Cellular Phone") OR (MH "Smartphone") OR(MH "Text Messaging") | 8,244 |
| 16 | (MH "Algorithms") | 40,446 |
| 17 | (MH "Artificial Intelligence+") | 21,256 |
| 18 | (MH "Expert Systems") | 529 |
| 19 | (MH "Knowledge Bases") | 683 |
| 20 | (MH "Machine Learning+") | 2,948 |
| 21 | (MH "Natural Language Processing") | 2,190 |
| 22 | (MH "Neural Networks (Computer)") | 2,757 |
| 23 | (MH "Social Media+") | 17,921 |
| 24 | (MM "Internet") OR (MM "World Wide Web") | 41,170 |
| 25 | ((mobile or online or web or internet) N3 (application* or app or apps or interface* or platform* or program* or tech* or intervention*)) | 33,871 |
| 26 | smartphone* or smartphone* or tablet orcellphone* or cell phone* or mobile phone* or iphone* or android* | 25,791 |
| 27 | Text messag* | 4,971 |
| 28 | e-health or ehealth or m-health or mhealth | 5,963 |
| 29 | social media* or social network* site* or social network* website* or facebook* or instagram* or snapchat* or Linkedin* or Weibo* or Whatsapp* or Whats app* or Telegram* or WeChat* orWe Chat* or tiktok* | 27,860 |
| 30 | ((twitter* or tweet*) not tweetable abstract) | 4,774 |
| 31 | (forum* N3 (internet or web* or chat*)) | 525 |
| 32 | patientsLikeMe | 45 |
| 33 | wiki* | 962 |
| 34 | wiki* | 962 |
| 35 | algorithm* | 63,267 |
| 36 | (artificial intelligence or "AI" or "A.I.") | 19,550 |
| 37 | computational intelligence | 31 |
| 38 | machine learning or deeplearning or natural language processing | 12,641 |
| 39 | information N3 technolog* | 25,658 |
| 40 | communication* N3 technolog* | 3,955 |
| 41 | programming language* | 917 |
| 42 | software design* | 3,997 |
| 43 | user-centered design* | 405 |
| 44 | user-computer interface* | 10,846 |
| 45 | web browser* or wordprocess* | 1,598 |
| 46 | digital technolog* | 1,854 |
| 47 | ((computer* or digital* or virtual*) N3 simulat*) | 20,203 |
| 48 | virtual realit* | 8,400 |
| 49 | video gam* | 5,871 |
| 50 | wireless technolog* | 300 |
| 51 | expert system* | 846 |
| 52 | fuzzy logic | 244 |
| 53 | neural network* | 6,012 |
| 54 | (MH "Suicide") OR (MH" Suicidal Ideation") OR(MH "Suicide, Attempted") | 30,540 |
| 55 | (MH "Self-Injurious Behavior") | 4,503 |
| 56 | (MH "Injuries, Self-Inflicted") | 2,785 |
| 57 | (suicid* N3 prevent*) | 9,534 |
| 58 | (suicid* N3 (attempt* or commit* or complet* or die* or dead or ideation* or thought* or plan* or consider* or contemplat*or behavio?r* or method*)) | 21,568 |
| 59 | suicidal* | 17,629 |
| 60 | (selfharm* or self-harm or self injur* or self-injur* or selfinflict* or self-inflict* or self-mutilat* or selfmutilat* or selfpoison* or self-poison* or automutilat*) | 10,181 |
| 61 | ((fatal* or lethal* or intentional* or deliberate*) N2 (dose or doses or dosing or overdos* or self-administ* or selfadminist*)) | 1,514 |
| 62 | (MH "Practice Patterns") | 12,867 |
| 63 | (MH "Health Care Delivery") | 56,945 |
| 64 | (MH "Health Care Delivery, Integrated") | 12,827 |
| 65 | (MH "Health Services Accessibility+") | 94,057 |
| 66 | (MH "Telemedicine+") | 16,547 |
| 67 | (MH "Health Facilities")OR (MH "Academic Medical Centers") OR (MH "Ambulatory CareFacilities+") OR (MH"Community Health Centers+") OR (MH"Health Facility Departments+") OR (MH"Hospital Units+") OR (MH "Hospitals+") OR (MH "Rehabilitation Centers+") OR (MH"Residential Facilities+") | 446,656 |
| 68 | TI ( (clinical* N3 (practic* or practis* or application* or care)) ) OR AB ((clinical* N3 (practic* orpractis* or application* or care)) ) OR MW ( (clinical*N3 (practic* or practis* or application* or care)) ) | 135,617 |
| 69 | ((health or healthcare or medical or psychiatr* or mental* or rehab* or treatment* or inpatient* or outpatient* or walk-in or drop-in) N3 (hospital* or institut* or setting* or environment* or clinic* or centre* or center* or facility or facilities orward* or unit* or office* or program* or service* or intervention*)) | 958,611 |
| 70 | (clinical N3 (hospital* or institut* or setting* or environment* or centre* or center* or facility or facilities or ward* or unit* or office* or program* or service* or intervention*)) | 86,762 |
| 71 | (care team* or healthcareteam* or health team*) | 59,036 |
| 72 | ((health* or healthcare or medical* or mental* or psychiatr* or inpatient* or outpatient*) N3 (session* or appointment*)) | 4,363 |
| 73 | ((nurse* or nursing) N3 (hospital* or institut* ors etting* or environment* or clinic* or centre* or center* or facility or facilities or ward* or unit* or office* or program* or service* or intervention* or care)) | 296,493 |
| 74 | medical home* | 3,066 |
| 75 | hospital* | 598,581 |
| 76 | (emergency N3 (department* or ward* or unit* or room* or service* or medicine or center* or centre* or clinic or clinics or hospital* or care or visit* or patient*)) | 164,071 |
| 77 | (casualty N1 (department* or ward* or unit* or room* or service* or medicine or center* or centre* or clinic or clinics or hospital* or visit* or patient*)) | 295 |
| 78 | a&e | 6,738 |
| 79 | TI ("ER" or "E.R.") | 3,291 |
| 80 | (telemental* or telemental* or teletherap* or telepsych* or telemedic* or telehealth* or teleconferenc* or tele-psychotherap* or tele-psychiatr* or tele-medic* or tele-health* or tele-conferenc*) | 30,026 |
| 81 | ((health* or healthcare or medical* or mental* or psychiatr*) N3(profession* or work* or practitioner* or provider* or clinician* or servic*)) | 552,306 |
| 82 | (physician* or doctor* or primary care or general practitioner* or nurse practitioner*) | 392,440 |
| 83 | (psychiatrist* or psychologist*) | 20,788 |
| 84 | ((health* or medic* or clinic*) N3 (aide* or assistant* or technician* or navigator*)) | 22,728 |
| 85 | (peer* N3 (worker* or coach* or navigator*)) | 863 |
| 86 | (digital N3 (coach* or navigator*)) | 44 |
| 87 | ((regulated or unregulated) N3 (provider* or professional* or worker*)) | 217 |
| 88 | TI ( (treatment* or intervention*) ) OR MW ((treatment* or intervention*) ) | 744,920 |
| 89 | (allerg* or immunolog* or anesthesiolog* or dermatolog* or radiolog*or emergency medicine or family medicine or internal medicine or internist* or neurology* or obstetric* or gynecolog* or ophthalmolog* or pathology or pathologist* or pediatric* or paediatric* or oncolog* or surgeon* or surgical* or surgery or urolog*) | 1,549,243 |
| 90 | S1 OR S2 OR S3 OR S4 OR S5 OR S6 OR S7 OR S8 OR S9 OR S10 OR S11 OR S12 OR S13 OR S14 OR S15 OR S16 OR S17 OR S18 OR S19 OR S20 OR S21 OR S22 OR S23 OR S24 OR S25 OR S26 OR S27 OR S28 OR S29 OR S30 OR S31 OR S32 OR S33 OR S34 OR S35 OR S36 OR S37 OR S38 OR S39 OR S40 OR S41 OR S42 OR S43 OR S44 OR S45 OR S46 OR S47 OR S48 OR S49 OR S50 OR S51 OR S52 OR S53 | 297,965 |
| 91 | S54 OR S55 OR S56 OR S57 OR S58 OR S59 OR S60 OR S61 | 44,888 |
| 92 | S62 OR S63 OR S64 OR S65 OR S66 OR S67 OR S68 OR S69 OR S70 OR S71 OR S72 OR S73 ORS74 OR S75 OR S76 ORS77 OR S78 OR S79 OR S80 OR S81 OR S82 OR S83 OR S84 OR S85 ORS86 OR S87 OR S88 | 2,646,566 |
| 93 | S90 AND S91 AND S92 | 746 |

Web of Science Core Collection – August 20, 2021

| Line | Query | Records Retrieved |
| --- | --- | --- |
| 1 | ((mobile or online or web or internet) NEAR/3 (application* or app or apps or interface* or platform* or program* or tech* or intervention*)) (Topic) | 229958 |
| 2 | TS=((smartphone* or "smart phone*" or tablet* or cellphone* or "cell phone*" or "mobile phone*" or iphone* or android*)) | 199606 |
| 3 | "Text messag*" (Topic) | 8020 |
| 4 | "e-health" or ehealth or "m-health" or mhealth (Topic) | 22487 |
| 5 | ("social media*" or "social network* site*" or "social network* website*" or facebook* or instagram* or snapchat* or Linkedin* or Weibo* or Whatsapp* or "Whats app*" or Telegram* or WeChat* or "We Chat*" or tiktok*). (Topic) | 104264 |
| 6 | ((twitter* or tweet*) not "tweetable abstract"). (Topic) | 30982 |
| 7 | (forum* NEAR/3 (internet or web* or chat*)) (Topic) | 2876 |
| 8 | PatientsLikeMe (Topic) | 105 |
| 9 | wiki* (Topic) | 12324 |
| 10 | algorithm* (Topic) | 2269706 |
| 11 | ("artificial intelligence" or "AI" or "A.I.") (Topic) | 167654 |
| 12 | "computational intelligence" (Topic) | 6629 |
| 13 | ("machine learning" or "deep learning" or "natural language processing") (Topic) | 279704 |
| 14 | information NEAR/3 technolog* (Topic) | 152267 |
| 15 | communication NEAR/3 technolog* (Topic) | 76133 |
| 16 | #1 or #2 or #3 or #4 or #5 or #6 or #7 or #8 or #9 or #10 or #11 or #12 or #13 or #14 or #15 | 3193237 |
| 17 | suicid* NEAR/3 prevent* (Topic) | 9722 |
| 18 | TS=((suicid* NEAR/3 (attempt* or commit* or complet* or die* or dead or ideation* or thought* or plan* or consider* or contemplat* or behavior* or behaviour* or method*))) | 50577 |
| 19 | suicidal* (Topic) | 42800 |
| 20 | (selfharm* or "self-harm*" or selfinjur* or "self-injur*" or selfinflict* or "self-inflict*" or "self-mutilat*" or selfmutilat* or selfpoison* or "self-poison*" or automutilat* OR "auto-mutilat*") (Topic) | 23089 |
| 21 | ((fatal* or lethal* or intentional* or deliberate*) NEAR/2 (dose or doses or dosing or overdos* or self-administ* or selfadminist*)) (Topic) | 13525 |
| 22 | #17 OR #18 OR #19 OR #20 OR #21 | 92915 |
| 23 | TS=((clinical* NEAR/3 (practice* or practise* or application* or care))) | 395355 |
| 24 | TS=(((health or healthcare or medical or psychiatr* or mental* or rehab* or treatment* or inpatient* or outpatient* or "walk-in" or "drop-in") NEAR/3 (hospital* or institut* or setting* or environment* or clinic* or centre* or center* or facility or facilities or ward* or unit* or office* or program* or service* or intervention*))) | 1255083 |
| 25 | (clinical NEAR/3 (hospital* or institut* or setting* or environment* or centre* or center* or facility or facilities or ward* or unit* or office* or program* or service* or intervention*)) (Topic) | 208140 |
| 26 | "care team*" or "healthcare team*" or "health team" (Topic) | 17084 |
| 27 | ((health* or healthcare or medical* or mental* or psychiatr* or inpatient* or outpatient*) NEAR/3 (session* or appointment*)) (Topic) | 7927 |
| 28 | ((nurse* or nursing) NEAR/3 (hospital* or institut* or setting* or environment* or clinic* or centre* or center* or facility or facilities or ward* or unit* or office* or program* or service* or intervention* or care)) (Topic) | 123563 |
| 29 | "medical home*" (Topic) | 4579 |
| 30 | hospital* (Topic) | 1298550 |
| 31 | (emergency NEAR/3 (department* or ward* or unit* or room* or service* or medicine or center* or centre* or clinic or clinics or hospital* or care or visit* or patient*)) (Topic) | 201715 |
| 32 | (casualty NEAR/1 (department* or ward* or unit* or room* or service* or medicine or center* or centre* or clinic or clinics or hospital* or visit* or patient*)) (Topic) | 872 |
| 33 | "a&e" (Topic) | 19209 |
| 34 | ("ER" or "E.R.") (Title) | 39060 |
| 35 | (telemental* or "tele mental*" or teletherap* or telepsych* or telemedic* or telehealth* or "teleconferenc*" or "tele-psychotherap*" or "tele-psychiatr*" or "tele-medic*" or "tele-health*" or "tele-conferenc*") (Topic) | 39059 |
| 36 | ((health* or healthcare or medical* or mental* or psychiatr*) NEAR/3 (profession* or work* or practitioner* or provider* or clinician* or servic*)) (Topic) | 567616 |
| 37 | (physician* or doctor* or "primary care" or "general practitioner*" or "nurse practitioner*") (Topic) | 657089 |
| 38 | psychiatrist* or psychologist* (Topic) | 63421 |
| 39 | ((health* or medic* or clinic*) NEAR/3 (aide* or assistant* or technician* or navigator*)) (Topic) | 9653 |
| 40 | (peer* NEAR/3 (worker* or coach* or navigator*)) (Topic) | 1770 |
| 41 | (digital NEAR/3 (coach* or navigator*)) (Topic) | 162 |
| 42 | ((regulated or unregulated) NEAR/3 (provider* or professional* or worker*)) (Topic) | 999 |
| 43 | treatment* or intervention* (Title) or treatment* or intervention* (Author Keywords) | 1982708 |
| 44 | (allerg* or immunolog* or anesthesiolog* or dermatolog* or radiolog* or "emergency medicine" or "family medicine" or "internal medicine" or internist* or neurology* or obstetric* or gynecolog* or ophthalmolog* or pathology or pathologist* or pediatric* or paediatric* or oncolog* or surgeon* or surgical* or surgery or urolog*) (Topic) | 3743561 |
| 45 | ("social work*" or "occupational therap*" or "allied health*" or pharmacy or pharmacist* or physiotherap* or dentist* or dental* or audiolog* or "speech patholog*" or "language patholog*" or chiropod* or podiatr* or chiropract* or dentur* or dietician* or dietetic* or homeopath* or naturopath* or kinesiolog* or "massage therap*" or midwif* or midwiv* or optician* or optometr* or psychotherap* or "psycho-therap*" or "respiratory therap*" or "chinese medicine" or acupunctur* or (laborator* NEAR/3 (technolog* or technician*)) or ((radiation or radiolog*) NEAR/3 (technolog* or technician*))) (Topic) | 665399 |
| 46 | #23 or #24 or #25 or #26 or #27 or #28 or #29 or #30 or #31 or #32 or #33 or #34 or #35 or #36 or #37 or #38 or #39 or #40 or #41 or #42 or #43 or #44 or #45 | 8221891 |
| 47 | #16 AND #22 AND #46 | 1307 |
| 48 | #16 AND #22 AND #46 and Book Chapters or Meeting Abstracts (Exclude – Document Types) | 1282 |

Library, Information Science and Technology Abstracts (LISTA) – August 20, 2021

| Line | Query | Records Retrieved |
| --- | --- | --- |
| 1 | suicid* N3 prevent* | 134 |
| 2 | (suicid* N3 (attempt* or commit* or complet* or die* or dead or ideation* or thought* or plan* or consider* or contemplat* or behavio?r* or method*)) | 285 |
| 3 | suicidal* | 359 |
| 4 | (selfharm* or self-harm or selfinjur* or self-injur* or selfinflict* or self-inflict* or self-mutilat* or selfmutilat*or selfpoison* or self-poison* or automutilat*) | 157 |
| 5 | ((fatal* or lethal* or intentional* or deliberate*) N2 (dose or doses or dosing or overdos* or self-administ* or selfadminist*)) | 17 |
| 6 | S1 OR S2 OR S3 OR S4 OR S5 | 764 |
| 7 | (clinical* N3 (practic* or practis* or application* or care)) | 1,769 |
| 8 | ((health or healthcare or medical or psychiatr* or mental* or rehab* or treatment* or inpatient* or outpatient* or walk-in or drop-in) N3 (hospital* or institut* or setting* or environment* or clinic* or centre* or center* or facility or facilities or ward* or unit* or office* or program* or service* or intervention*)) | 34,360 |
| 9 | (clinical N3 (hospital* or institut* or setting* or environment* or centre* or center* or facility or facilities or ward* or unit* or office* or program* or service* or intervention*)) | 2,450 |
| 10 | care team* or healthcare team* or health team | 467 |
| 11 | ((health* or healthcare or medical* or mental* or psychiatr* or inpatient* or outpatient*) N3 (session*or appointment*)) | 212 |
| 12 | ((nurse* or nursing) N3 (hospital* or institut* or setting* or environment* or clinic* or centre* or center* or facility or facilities or ward* or unit* or office* or program* or service* or intervention* or care)) | 2,098 |
| 13 | medical home* | 91 |
| 14 | hospital* | 15,030 |
| 15 | (emergency N3 (department* or ward* or unit* or room* or service* or medicine or center* or centre* or clinic or clinics or hospital* or care or visit* or patient*)) | 1,418 |
| 16 | (casualty N1 (department* or ward* or unit* or room* or service* or medicine or center* or centre* or clinic or clinics or hospital* or visit* or patient*)) | 3 |
| 17 | a&e | 145,249 |
| 18 | TI "ER" or "E.R." | 210 |
| 19 | (telemental* or telemental* or teletherap* or telepsych* or telemedic*or telehealth* or teleconferenc* or tele-psychotherap* or tele-psychiatr* or tele-medic*or tele-health* or tele-conferenc*) | 2,497 |
| 20 | ((health* or healthcare or medical* or mental* or psychiatr*) N3 (profession* or work* or practitioner* or provider* or clinician* or servic*)) | 23,363 |
| 21 | (physician* or doctor* or primary care or general practitioner* or nurse practitioner*) | 17,121 |
| 22 | (psychiatrist* or psychologist*) | 1,508 |
| 23 | ((health* or medic* or clinic*) N3 (aide* or assistant* or technician* or navigator*)) | 538 |
| 24 | (peer* N3 (worker* or coach* or navigator*)) | 47 |
| 25 | (digital N3 (coach* or navigator*)) | 20 |
| 26 | ((regulated or unregulated) N3 (provider* or professional* or worker*)) | 6 |
| 27 | TI ( (treatment* or intervention*) ) OR KW ((treatment* or intervention*) ) | 4,766 |
| 28 | (allerg* or immunolog* or anesthesiolog* or dermatolog* or radiolog*or emergency medicine or family medicine or internal medicine or internist* or neurology* or obstetric* or gynecolog* or ophthalmolog* or pathology or pathologist* or pediatric* or paediatric* or oncolog* or surgeon* or surgical* or surgery or urolog*) | 12,454 |
| 29 | S7 OR S8 OR S9 OR S10 OR S11 OR S12 OR S13 OR S14 OR S15 OR S16 OR S17 OR S18 OR S19 OR S20 OR S21 OR S22 OR S23 OR S24 OR S25 OR S26 OR S27 OR S28 | 207,247 |
| 30 | S6 AND S29 | 199 |
