# Supplementary material for: Information and communication technology-based interventions for suicide prevention implemented in clinical settings: a scoping review
Source: BMC Health Serv Res. 2023 Mar 23;23:281. doi: 10.1186/s12913-023-09254-5 (PMC10037806; doi:10.1186/s12913-023-09254-5)
Supplement: Supplementary file 2 — Additional file 2. [file 12913_2023_9254_MOESM2_ESM.docx]

Additional File 2. Data extraction tool

| Title |  |
| --- | --- |
| Reviewer name |  |
| Title |  |
| Year |  |
| Authors |  |
| Country of origin |  |
| Study design |  |
| Aim/objectives/research questions of study |  |
| Theory/model/framework |  |
| Study setting |  |
| Description of clinical setting(s) |  |
| Type of clinical setting(s) | Select all   - In-patient - Out-patient - Mixture - Undetermined |
| Geography |  |
| Digital equity |  |
| Inclusion criteria |  |
| Population description (demographics, age, etc.) |  |
| Total number of participants |  |
| Clinician description (demographics, age) |  |
| Discipline (physician, nurses, social workers, etc.) |  |
| Total number of clinicians |  |
| Implementation strategy(s) description |  |
| Intervention target population (can select both) | - Adult - Youth |
| ICT intervention name(s) |  |
| ICT intervention description |  |
| Reported barriers |  |
| Reported facilitators |  |
| Outcomes of interest (description) |  |
| Measurement tool (e.g., survey, scale) |  |
| Reported outcomes |  |
