# Supplementary material for: Information and communication technology-based interventions for suicide prevention implemented in clinical settings: a scoping review
Source: BMC Health Serv Res. 2023 Mar 23;23:281. doi: 10.1186/s12913-023-09254-5 (PMC10037806; doi:10.1186/s12913-023-09254-5)
Supplement: Supplementary file 3 — Additional file 3. [file 12913_2023_9254_MOESM3_ESM.docx]

Additional fie 3. Full coding strategy

Use **Bolded** for coding

| Mental Health Commissions Canada (MHCC) typology for ICTs [37] | |
| --- | --- |
| Typology | Example |
| **Computerized interventions, resources, and applications** | “Computerized interventions deliver services directly to patients with or without the  aid of a person.”  Web-based tools, texting, applications (apps) |
| **Telemedicine or Telehealth** | Telephone-based |
| **Wearable computing and monitoring** | Wearable computing device, as well as apps that monitor activities, physiology, and habits. They can “provide sophisticated, often real time data to both the patient and clinicians about important outcomes.”  Fitbit, Google glass |
| **Big data** | “The use of large amounts of data to predict future behaviour and outcomes.” “Examples in mental health include the use of data from individual health providers on who responds to what treatment to predict what individual  patients should be offered in the future.” |
| **Virtual reality** | “Computer generated simulation of a three-dimensional environment” |
| **Peer support through social media and other technologies** | Big White Wall, Mindyourmind  “Mindyourmind is a London, Ontario-based online support forum for youth, emerging  adults, and professionals. The focus is to use youth networks to provide a platform for  youth to express their views around mental health, and to support the production of  useful resources for youth with mental illness.” |
| **Robots** | Therapeutic robots that mimic animals, such as Paro. |
| **Gaming** | “Sparx a game based on an imaginary island where participants go on a quest and have to fight negative automatic thoughts and overcome problems to progress.” |

| Suicide Prevention Intervention Category [38,39] | |
| --- | --- |
| Type | Example(s) |
| **Screening and assessment** | Screening tools  Risk assessment tools |
| **Safety planning** | Identifying warming signs and/or coping strategies, emergency contacts |
| **Lethal means restrictions and/or counselling** | Assessing for access to firearms, and/or how to safely store them |
| **Discharge or post-discharge follow up** | Follow-up calls |
| **Therapy** | Behavioural Therapies |
| **Resources** | Other supportive tools for patients |

| WHO Suicide Prevention Strategy [1] | |
| --- | --- |
| Category | Definition |
| **Universal prevention strategy** | Designed to reach an entire population in an effort to maximize health and minimize suicide risk by removing barriers to care and increasing access to help, strengthening protective processes such as social support and altering the physical environment. |
| **Selective prevention strategy** | Target vulnerable groups within a population based on characteristics such as age, sex, occupational status or family history. While individuals may not currently express suicidal behaviours, they may be at an elevated level of biological, psychological or socioeconomic risk. |
| **Indicated prevention strategy** | Target specific vulnerable individuals within the population − e.g. those displaying early signs of suicide potential or who have made a suicide attempt. |

| Barriers and Facilitators | | |
| --- | --- | --- |
| COM-B component  (Definition)  [42, 42] | TDF Version 2 [43] | |
|  | 14 Domains (Definition) | Constructs |
| **Capability**  (Capability is defined as the individual's psychological and physical capacity to engage in the activity concerned) | **Knowledge** (An awareness of the existence of something) | Knowledge (including knowledge of condition/scientific rationale) Procedural knowledge Knowledge of task environment |
|  | **Skills** (An ability or proficiency acquired through practice) | Skills Skills development Competence Ability Interpersonal skills Practice Skill assessment |
|  | **Memory, attention and decision processes** (The ability to retain information, focus selectively on aspects of the environment and choose between two or more alternatives) | Memory Attention Attention control Decision making Cognitive overload/tiredness |
|  | **Behavioural regulation** (Anything aimed at managing or changing objectively observed or measured actions) | Self-monitoring Breaking habit Action planning |
| **Motivation**  (Motivation is defined as all those  brain processes that energize and direct behaviour, not  just goals and conscious decision-making. It includes  habitual processes, emotional responding, as well as analytical decision-making.) | **Social/professional role and identity** (A coherent set of behaviours and displayed personal qualities of an individual in a social or work setting) | Professional identity Professional role Social identity Identity Professional boundaries Professional confidence Group identity Leadership Organisational commitment |
|  | **Beliefs about capabilities** (Acceptance of the truth, reality or validity about an ability, talent or facility that a person can put to constructive use) | Self-confidence Perceived competence Self-efficacy Perceived behavioural control Beliefs Self-esteem Empowerment Professional confidence |
|  | **Optimism** (The confidence that things will happen for the best or that desired goals will be attained) | Optimism Pessimism Unrealistic optimism Identity |
|  | **Beliefs about Consequences** (Acceptance of the truth, reality, or validity about outcomes of a behaviour in a given situation) | Beliefs Outcome expectancies Characteristics of outcome expectancies Anticipated regret Consequents |
|  | **Reinforcement** (Increasing the probability of a response by arranging a dependent relationship, or contingency, between the response and a given stimulus) | Rewards (proximal/distal, valued/not valued, probable/improbable) Incentives Punishment Consequents Reinforcement Contingencies Sanctions |
|  | **Intentions** (A conscious decision to perform a behaviour or a resolve to act in a certain way) | Stability of intentions Stages of change model Transtheoretical model and stages of change |
|  | **Goals** (Mental representations of outcomes or end states that an individual wants to achieve) | Goals (distal/proximal) Goal priority Goal/target setting Goals (autonomous/controlled) Action planning Implementation intention |
|  | **Emotion** (A complex reaction pattern, involving experiential, behavioural, and physiological elements, by which the individual attempts to deal with a personally significant matter or event) | Fear Anxiety Affect Stress Depression Positive/negative affect Burn-out |
| **Opportunity**  (Opportunity is defined as all the  factors that lie outside the individual that make the  behaviour possible or prompt it) | **Environmental context and resources** (Any circumstance of a person’s situation or environment that discourages or encourages the development of skills and abilities, independence, social competence and adaptive behaviour) | Environmental stressors Resources/material resources Organisational culture/climate Salient events/critical incidents Person × environment interaction Barriers and facilitators |
|  | **Social influences** (Those interpersonal processes that can cause individuals to change their thoughts, feelings, or behaviours) | Social pressure Social norms Group conformity Social comparisons Group norms Social support Power Intergroup conflict Alienation Group identity Modelling |

| Outcome Measures [48-51] | | | |
| --- | --- | --- | --- |
| Dimension (Definition) | Level | Domains | Example |
| **Effectiveness**  (Impact of an intervention on outcomes, including potential negative effects, quality of life, and economic outcomes) | **Health care provider** | **Conceptual knowledge use** (proximal behaviour change) | - Knowledge - Attitude - Self-efficacy |
|  |  | **Instrumental knowledge use** (observable behaviour change) | - Rates of referrals - Rates of completed assessments - Adherence to clinical practice guideline |
|  | **Patient** | **Patient reported outcomes or experience** (PRO or PRE)  A measurement based on a report that comes directly from the patient (i.e., study subject) about the status of a patient’s health condition without amendment or interpretation of the patient’s response by a clinician or anyone else. A PRO can be measured by self-report or by interview provided that the interviewer records only the patient’s response.  PRO: Perceptions of their health status, perceived level of impairment, disability, and health-related quality of life  PRE: patients’ views of their experience whilst receiving care. They are an indicator of the quality of patient care, | - Symptoms (e.g., pain) - Function - Quality of life - Patient satisfaction |
|  |  | **Patient health outcomes** | - Mortality - Morbidity - Physiological measures |
|  | **Health system** | **Resource utilization, coverage, access, use** | - Admission rates - Readmission - Length of stay - Waiting times to see a doctor - Cost analysis |
| **Implementation Outcomes**  **(**The intervention agents’ fidelity to the various elements of an intervention’s protocol) | **Acceptability**  Acceptability is the perception among implementation stakeholders that a given treatment, service, practice, or  innovation is agreeable, palatable, or satisfactory. | | |
|  | **Adoption**  Adoption is defined as the intention, initial decision, or action to try or employ an innovation or evidence-based practice. Adoption also may be referred to as ‘‘uptake.’’ | | |
|  | **Appropriateness**  Appropriateness is the perceived fit, relevance, or compatibility of the innovation or evidence-based practice for a given practice setting, provider, or consumer; and/or perceived fit of the innovation to address a particular issue or problem. ‘‘Appropriateness’’ is conceptually similar to ‘‘acceptability,’: Distinction because a given treatment may be perceived as appropriate but not acceptable, and vice versa. | | |
|  | **Feasibility**  Feasibility is defined as the extent to which a new treatment, or an innovation, can be successfully used or carried out within a given agency or setting. While feasibility is related to appropriateness, the two constructs are conceptually distinct. For example, a program may be appropriate for a service setting—in that it is compatible with the setting’s mission or service mandate, but may not be feasible due to resource or training requirements. | | |
|  | **Fidelity**  Fidelity is defined as the degree to which an intervention was implemented as it was prescribed in the original protocol or as it was intended by the program developers. The literature identifies five implementation fidelity dimensions including adherence, quality of delivery, program component differentiation, exposure to the intervention, and participant responsiveness or involvement | | |
|  | **Implementation cost**  Cost (incremental or implementation cost) is defined as the cost impact of an implementation effort. | | |
|  | **Penetration**  Penetration is defined as the integration of a practice within a service setting and its subsystems | | |
|  | **Sustainability**  Sustainability is defined as the extent to which a newly implemented treatment is maintained or institutionalized within a service setting’s ongoing, stable operations. | | |
