# Supplementary material for: Information and communication technology-based interventions for suicide prevention implemented in clinical settings: a scoping review
Source: BMC Health Serv Res. 2023 Mar 23;23:281. doi: 10.1186/s12913-023-09254-5 (PMC10037806; doi:10.1186/s12913-023-09254-5)
Supplement: Supplementary file 4 — Additional file 4. [file 12913_2023_9254_MOESM4_ESM.docx]

| **Author, Year** | **Capability** | **Motivation** | | **Opportunity** | |
| --- | --- | --- | --- | --- | --- |
|  | **Psychological** | **Reflective** | **Autonomic** | **Physical** | **Social** |
| Brand et al., 2021 | No access to appropriate training to ensure that nurses feel able to use innovative technology (Barrier) | NR | NR | Patients' lack of access to the technology required to operate the True Colours system (Barrier) | NR |
| Bruen et al., 2020 | NR | Concerned for who would be responsible for monitoring the devices (Barrier)  Concerns about giving service users iPhones and Fitbits for the duration of the study, suggesting that the equipment would either be stolen or damaged (Barrier)  Appropriate professional role of staffs to identify eligible patients for the intervention (Facilitator) | NR | No access to the research team available in participating wards to troubleshoot technological issues in a timely manner (Barrier) | Positive working collaborations between clinicians and the research team, including data scientists and technicians, to ensure a continuous flow of data (Facilitator) |
| Bush et al., 2015 | Access to detailed downloadable user guides for clinicians and patients (Facilitator) | Perceived benefit of the intervention (Facilitator) | NR | NR | NR |
| Dimeff et al.,2020 | Available training materials about how to do specific CAMS and other EBP therapeutic tasks. (Facilitator)  Clinical decision support tool to provide a definitive recommendation about whether to hospitalize or release a patient, decreasing cognitive burden (Facilitators) | NR | Providers viewed Virtual CAMS integration into their electronic health records as essential requirement going forward (Facilitator) | NR | NR |
| Gregory et al., 2017 | NR | NR | NR | Patients cannot use phone until discharge (Barrier) | Waiting to download the app until the moment of discharge limits the opportunity for staff to facilitate the adoption of a smartphone app (Barrier) |
| Gros et al., 2011 | NR | NR | NR | Resources that are tied up the only line of communication between patient and provider (Barrier) | NR |
| Kasckow et al., 2015 | NR | When implementing the intervention, the burden on staff time is not appreciable (Barrier) | NR | Limited staff time (Barrier) | NR |
| Kroll et al., 2020 | NR | NR | NR | No clear instructions in the protocol which led to protocol violation, in which a patient with suicide risk on a non-pilot unit was placed on virtual monitoring (Barrier) | NR |
| Luxton et al., 2012 | NR | NR | NR | NR | Usual discharge practice limited opportunity to deliver ICT to patients (Barrier) |
| Luxton et al., 2014 | NR | NR | NR | Inexpensive ICT (Facilitator) | Having a hospital staff member in the role of principal investigator at each site (Facilitator) |
| Luxton et al., 2020 | NR | Task can be reasonably done by existing hospital staff and the minimal requirement to manage replies from participants who were in crisis (Facilitator) | NR | NR | NR |
| Mackie et al., 2017 | NR | ICT did not fully integrate with the face-to-face therapy (Barrier)  Perceived usefulness to send reminders and this increased the sense of connection between the patients and therapist (Facilitator)  Perceived advantage of adding extra resources relevant to discussion points raised during face-to-face treatment or information for friends and family on how to manage someone who was cutting (Facilitator) | Using the smartphone application was ‘some-thing else to talk about’ and became like a ‘third person’ in the room (Barrier) | Using the smartphone application was more time consuming (Barrier) | Increased the sense of connection between the patients and therapist (Facilitator) |
| Madan et al., 2015 | NR | NR | NR | Administrative challenges: reported need for protocols that support social accountability for patient safety (Barrier) | NR |
| Muscara et al., 2020 | NR | NR | NR | NR | Limited engagement of clinicians in the study (Barrier) |
| O'Toole et al., 2019 | No manual or guidelines as to how the mobile app should be introduced and used throughout treatment (Barrier) | Causing extra work for the usual therapy (Barrier)  Uncertainty about how well incorporated the mobile app was in the face-to-face treatment, and whether this led to a positive or negative effect (Barrier) | NR | Taking extra time away from the usual therapy (Barrier) | NR |
| Owens et al., 2016 | The effort involved in mastering a new technology and incorporating it into everyday practice (Barrier) | Perception that the intervention may have better fit with schools and universal youth services (Barrier)  Clinicians quickly grasped the basic principles and saw it as a potentially valuable tool to help young people manage their self-harming behaviour (Facilitator) | Perceived burdensomeness and technophobia (Barrier)  Clinicians and managers agreed that the new ICT made sense and was immediately appealing (Facilitator) | With heavy workloads and high stress levels, organizational gatekeeping practices limited the extent to which clinicians could engage with the intervention (Barrier) | Limited buy-in at some management levels (Barrier)  Manager approvals for implementation and some managers insisted on circulating information to clinicians via e-mail and managing the recruitment process (Facilitator) |
| Parkland 2018 | Educating staff about the reasons for universal screening prior to implementation (Facilitator)  Solid understanding of existing institutional resources and community mental health resources (Facilitator) | Social workers do the vast majority of clinical responsibility in the program and their clinical skills help support this program (Facilitator) | NR | NR | Increase buy-in/institutional support (Facilitator) |
| Pickett et al., 2021 | NR | NR | NR | NR | Lack of buy-in from key stakeholders to simple logistics (Barrier) |
| Wright et al., 2021 | Training prepared health care professionals for assessing and caring for patients from a distance using mobile telehealth iPad interactions (Facilitator) | NR | NR | Occasional dropped or slow connections, pixel blurring, and the need for online security (Barrier) | NR |
| NR: Not reported | | | | | |
